# Non-metabolic role of UCK2 links EGFR-AKT pathway activation to metastasis enhancement in hepatocellular carcinoma

**DOI:** 10.1038/s41389-020-00287-7

**Published:** 2020-12-04

**Authors:** Jie Cai, Xuehua Sun, Han Guo, Xiaoye Qu, Hongting Huang, Chang Yu, Hailong Wu, Yueqiu Gao, Xiaoni Kong, Qiang Xia

**Affiliations:** 1 Department of Liver Surgery, Renji Hospital, School of Medicine, Shanghai Jiao Tong University, Shanghai, China; 2 Institute of Clinical Immunology, Department of Liver Diseases, Central Laboratory, ShuGuang Hospital Affiliated to Shanghai University of Chinese Traditional Medicine, Shanghai, China; 3 Shanghai Key Laboratory of Molecular Imaging, Collaborative Research Center, Shanghai University of Medicine & Health Sciences, Shanghai, China

**Keywords:** Liver cancer, Metastasis

## Abstract

Up-regulation of Uridine-cytidine kinase 2 (UCK2), a rate-limiting enzyme of the pyrimidine salvage pathway, has been suggested in HCC, but the detailed molecular mechanisms and therapic role of UCK2 remain elusive. Bioinformatic analyses revealed that UCK2 might be a key up-regulated metabolic gene in HCCs. The expressional pattern and prognostic value of UCK2 were further examined in a large number of clinical samples. Functional assays based on site-directed mutagenesis showed that UCK2 promoted cell proliferation in a metabolic manner, but non-catalytically facilitates HCC metastasis. Mechanistically, in response to EGF, UCK2 interacted with EGFR to block EGF-induced EGFR ubiquitination and degradation, which resulted in elevated EGFR-AKT pathway activation and metastasis enhancement in HCCs. Concurrent pharmacological targeting on UCK2 and EGFR showed synergistic effects on HCC treatment. This study disclosed the non-metabolic role of UCK2 and suggested the therapeutic potential of concurrent blocking the metabolic and non-metabolic roles of UCK2 in HCC treatment.

## Introduction

China reports over half of the new cases and deaths of liver cancer^10^. Hepatocellular carcinoma (HCC), which accounts for 85–90% of primary liver cancers, affects more than 700,000 patients every year^17^. Despite the multiple curative treatments available^29^, the prognosis is still poor in HCC patients mainly due to the high recurrence rate of HCC^18^, which is closely related to tumor invasion and metastasis. Therefore, identifying novel targets for effective treatment of HCC metastasis is urgently required.

Epidermal growth factor receptor (EGFR) is a transmembrane receptor tyrosine kinase, whose overexpression and activation have been frequently reported in many types of cancers^25^. Overexpression of EGFR has been found in 60–85% of HCCs and significantly correlated with advanced stage, intrahepatic and extrahepatic metastasis, poor differentiation and tumor recurrence^2,16,42^. Although genomic EGFR amplification and mutations have been reported to contribute to EGFR overexpression and activation in various types of cancers, including glioblastoma^14^, oligodendroglioma^11^, non-small-cell lung cancer^9^ and gastric carcinomas^32^, such genomic alterations of EGFR are barely reported in HCCs^8,13^, suggesting the existence of other mechanisms leading to the high frequency of EGFR overexpression and activation in HCCs.

Uridine-cytidine kinase (UCK) is a type of rate-limiting enzymes in the salvage pathway of pyrimidine-nucleotide biosynthesis. By mono-phosphorylation, UCK respectively converts cytidine and uridine to cytidine 5′-monophosphate and uridine 5′-monophosphate^4^. There are two UCK genes in humans, UCK1 and UCK2^38^. Despite the similarity of their catalytic functions, the catalytic efficacy of UCK2 in uridine and cytosine is 15–20 times higher than that of UCK1^38^, suggesting that UCK2 mainly contributes to the salvage pyrimidine-nucleotide biosynthesis. UCK2 up-regulation has been detected in several types of cancers including pancreatic tumor, colorectal cancer, neuroblastoma, breast cancer, and HCCs^28,37,40^. Some recent studies have demonstrated the correlation of UCK2 with poor prognosis of HCCs^40^, but the underlying mechanism by which UCK2 contributes to HCC progression and the metabolic/non-metabolic roles of UCK2 in HCCs are largely undetermined.

In present study, we demonstrate that UCK2 is highly expressed in HCC tissues and serves as an independent predictor of poor prognosis, negatively correlating with overall survival (OS), recurrence-free survival (RFS) and early-RFS (within 2 years). Functionally, both in vitro and in vivo assays showed that UCK2 promotes proliferation and metastasis of HCC cells. Interestingly, the kinase activity of UCK2 is required for UCK2-mediated proliferation but not for UCK2-induced metastasis in HCCs. Mechanistically, UCK2 interacts with EGFR to inhibit EGF-induced EGFR ubiquitination and degradation, resulting in increased EGFR stability and sustained activation of the EGFR-AKT pathway. Finally, we demonstrate the synergistic potential of concurrent pharmacological targeting on UCK2 and EGFR in HCC treatment in both xenograft and patient-derived xenograft (PDX) models.

## Materials and methods

### Patients and samples

Fresh human HCC tumor tissues and matched adjacent non-tumor liver tissues were collected from patients who underwent surgical resection at the Department of Transplantation and Hepatic Surgery, Renji Hospital, School of Medicine, Shanghai Jiao Tong University, between 2013 and 2017 for qRT-PCR (*n* = 120), western blot (*n* = 32), and immunohistochemistry (IHC) (*n* = 40) analyses. For clinical analyses, two independent TMA cohorts of HCC patients were retrospectively adopted. TMA cohort 1 included 153 HCCs collected between Feb 2004 and Aug 2008 from the Department of Transplantation and Hepatic Surgery, Renji Hospital, School of Medicine, Shanghai Jiao Tong University, and TMA cohort 2 included 307 HCCs collected between June 2007 and June 2012 from the Shanghai Eastern Hepatobiliary Surgery Hospital. Written informed consent was obtained from each patient, and the studies were approved by Shanghai Jiao tong University School of Medicine, Renji Hospital Ethics Committee.

### Establishment of patient-derived HCC cell lines

To establish the patient-derived HCC cell lines (PDC), fresh tumors were minced into ~1 mm^3^ fragments prior to enzymatic digestion using 1 mg/ml Collagenase type IV (17104019, Thermo Fisher, MA, USA) in DMEM/F12 (11320-033, Gibco, MA, USA), at 37 °C for 3 h. Then, cells were washed and re-suspended in phosphate buffered saline (PBS) (14190235, Thermo Fisher, MA, USA) for three cycles. The final cell suspensions were filtered with 70 µm cell-strainers (352350, Falcon, CA, USA) and centrifuged at 600 *g* for 5 min at 4 °C. The cell pellets were re-suspended in DMEM (12430-054, Gibco, MA, USA), supplemented with 10% (v/v) fetal bovine serum (FBS) (10099, Gibco, MA, USA), and 1% penicillin-streptomycin (15070063, Gibco, MA, USA) and cultured.

### Cell lines

The normal hepatocyte LO2 cells, as well as the commercially available HCC cell lines Huh-7, HepG2, HCC-LM3, SMMC-7721 and MHCC-97L, were purchased from the Shanghai Cell Bank of the Chinese Academy of Sciences (Shanghai, China). Home-made patient-derived HCC cell lines PDC-26#, PDC-14#, PDC-12#, PDC-9#, PDC-23#, PDC-10# and PDC-11# were established as described above. All of these cells were cultured in Dulbecco’s modified eagle medium (DMEM) supplemented with 10% (v/v) FBS and 1% penicillin-streptomycin at 37 °C in a humidified incubator under 5% CO_2_ condition.

### Phospho-antibody array

Huh-7 cells with stable overexpression of UCK2^WT^ or UCK2^D62A^, and control cells were gathered. The cell lysates were then obtained and applied to a Cancer Signaling Phospho-Antibody Array (PCS248, Full Moon Biosystems, CA, USA). The array experiment was performed by Wayen Biotechnologies (Shanghai, China) Inc. according to the manufacturer’s protocol. The array contained 269 site-specific and phospho-specific antibodies against total 93 proteins. Each specific site or protein contains six technical replicates. The slide was scanned on a GenePix 4000B scanner (Axon Instruments, CA, USA), and the images were analyzed with GenePix Pro 6.0. The fluorescence intensity of each array spot was quantified, and the mean value was calculated. The following equation was used to calculate a phosphorylation signal intensity (Phos-In): Phos-In = phospho A/unphospho A

where phospho A represented the signal from phosphorylated A protein, and unphospho A represented the signal from unphosphorylated A protein in the experimental samples.

### Proximity ligation assay

Proximity ligation assay was performed using the Duolink In Situ Red Starter Kit Mouse/Rabbit (Sigma-Aldrich, MO, USA). Briefly, cells were cultured on chamber slides, fixed with paraformaldehyde for 3 min. After washing, cells were permeabilized with 0.1% Triton X-100 in PBS for 5 min, blocked with PLA blocking buffer for 1 h and incubated with primary antibodies for overnight at 4 °C. Following three washes with PBS the cells were incubated with anti-rabbit-PLUS (Sigma-Aldrich; DUO92002 for UCK2 antibody) and anti-mouse-MINUS (Sigma-Aldrich; DUO92004 for EGFR antibody) PLA probes and subjected to ligation and amplification reaction using Duolink In Situ Detection Reagents Red (Sigma-Aldrich; DUO92008) according to the manufacturer’s protocol. The cells were mounted with DAPI and visualized with Leica DM IL LED-Inverted fluorescence microscope.

### Dose matrix experiments

For dose matrix experiments, cells were seeded in 96-well plates at 5 × 10^3^ cells per well. After 24 h, cells were treated with ECyd (dose range of 0–27 nM) and Gefitinib (dose range of 0–25 μM) in a 7 × 7 matrix. Cells were cultured with inhibitors for 72 h and cell viability was determined using CCK-8. Each treatment was done in triplicate wells. The following equation was used to calculate the Bliss Score: Bliss Score = AB − [(A + B) − A × B]. At a given dose, AB represented the fractional growth inhibitions induced by combined treatment with A and B, while A and B represented the fractional growth inhibitions induced by separate usage of A or B.

### Generation of PDXs

Tumor samples were obtained from patients post surgery after obtaining informed patient consent in accordance to the ethical review committee of the WHO Collaborating Center for Research in Human Production (authorized by Shanghai Municipal Government). Tumors were minced into ~1 mm^3^ fragments and suspended in a mixture of 5% Matrigel (356234, Corning, NY, USA) in DMEM/F12. The tumor fragment mixtures were then implanted subcutaneously into the left and right flanks of 5–7 weeks old NSG mice (Biocytogen, Beijing, China), using 18-gauge needles. Tumors were excised and passaged when they reached 1.5 cm^3^. For tumor passage, tissues were cut into small fragment of 1 mm^3^ prior to resuspension in 20% Matrigel/DMEM/F12 mix, before subcutaneous inoculation of tumor fragments into 5–7 weeks old BALB/C nude mice.

### Statistical analysis

Statistical analyses were performed with SPSS version 18.0 software. Graphical representations were performed with GraphPad Prism 6 software. Differentially expressed genes were obtained using online analysis tool GEO2R (https://www.ncbi.nlm.nih.gov/geo/geo2r/). The chi-square test or Fisher’s exact test were used to compare qualitative variables, and the Student’s *t* test or the Mann–Whitney test were used to compare continuous variables. Kaplan–Meier analysis was used to assess survival. Log-rank tests were used to compare survival of patients between subgroups. Multivariate analyses were performed by multivariate Cox proportional hazard regression model. Data were presented as mean ± SD. A difference was defined as significant at *p* < 0.05.

## Results

### Identification of UCK2 as a key up-regulated metabolic gene in HCCs

**Fig. 1:**
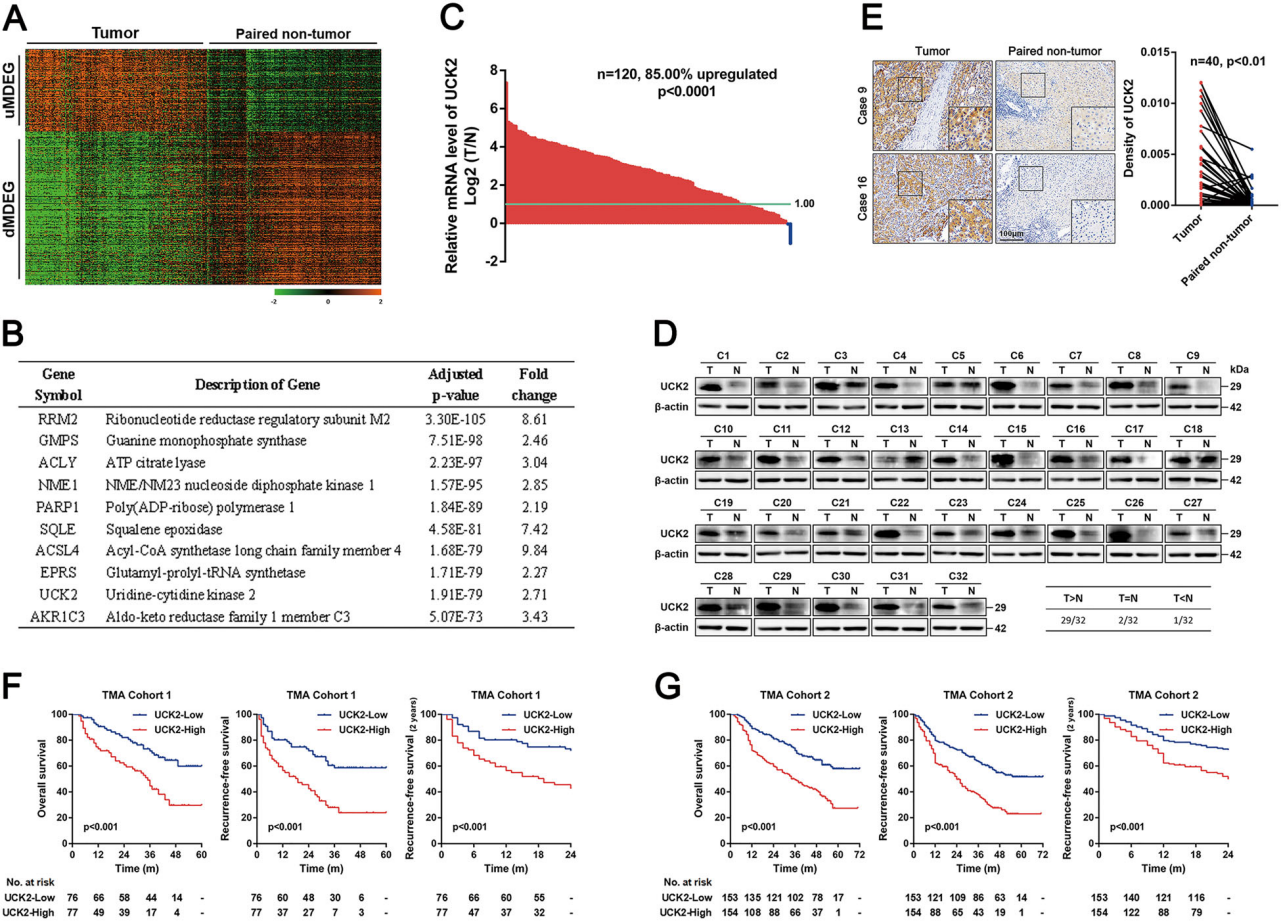
Identification of UCK2 as a key up-regulated metabolic gene in HCCs. **a** Heatmap of differentially expressed metabolic genes in GSE14520. **b** The top ten up-regulated metabolic genes in GSE14520 according to the *p* values. **c** qRT-PCR analyses of *UCK2* mRNA levels in 120 pairs of HCC tumor and adjacent non-tumor tissues. **d** UCK2 protein levels in 32 pairs of HCC tumor and adjacent non-tumor tissues detected by western blotting. **e** UCK2 protein levels in 40 pairs of HCC tumor and adjacent non-tumor tissues detected by IHC assays. **f**–**g** Kaplan–Meier analyses of overall survival (OS), recurrence-free survival (RFS) and early-RFS (within 2 years) of HCC patients in correlation with high or low UCK2 levels in TMA Cohort 1 (**f**) and TMA Cohort 2 (**g**). The absolute number of patients at risk is listed below each curve.

GSE14520, one HCC dataset in Gene Expression Omnibus (GEO) database that contains largest number of samples and detailed clinical prognostic information, was adopted to screen out dysregulated metabolic genes (MDEGs) in HCCs. Firstly, 2772 dysregulated genes [*p* value < 0.05, fold change (FC) > 1.5 or <−1.5] were identified by GEO2R, 62.2% of which were up-regulated and 37.8% down-regulated in HCC tissues (Fig. S1A). Meanwhile, 1151 genes assigned to metabolic pathways in the Kyoto Encyclopedia of Genes and Genomes database were gathered as metabolic genes (Fig. S1B). Finally, 155 up-regulated metabolic genes (uMDEGs) and 287 down-regulated metabolic genes were obtained by the overlapping analysis of these two gene sets (Fig. S1C). Heatmap assays according to these dysregulated metabolic genes apparently separated tumor and non-tumor tissues (Fig. 1A). Here, we are more interested in uMDEGs in HCCs. The top ten uMDEGs were listed according to the *p* values (Fig. 1B). Prognostic analyses showed that *UCK2* was the most effective uMDEG to differentiate the OS, RFS and early-RFS (within 2 years) of HCC patients in GSE14520 (Fig. S1D–S1F).

Consistent with the analyzed results and previous reports^40^, *UCK2* up-regulation in HCCs was confirmed in our great number of clinical samples (Fig. 1C–E). Furthermore, the prognostic value of *UCK2* in HCCs was confirmed in two independent HCC TMA cohorts (Fig. S1G and Table S1). Clinical association analyses revealed that increased *UCK2* levels were significantly associated several aggressive clinicopathological features (Table S2). Moreover, Kaplan–Meier analyses showed that UCK2 negatively correlates with OS, RFS and early-RFS in HCC patients (Fig. 1F, G), which was in line with the analyzed results of GSE14520. Multivariate Cox Regression showed that *UCK2* as an independent prognostic predictor for OS, RFS, and early-RFS (Table S3, 4). Furthermore, UCK2 also separated favorable and poor prognosis on OS, RFS and early-RFS in HCC patients at the same TNM stage (Fig. S1H–J). These findings indicate that UCK2 might be a key metabolic gene, which is commonly up-regulated and associated with poor prognosis in HCCs.

### *UCK2* promotes HCC proliferation, migration and invasion in vitro

**Fig. 2:**
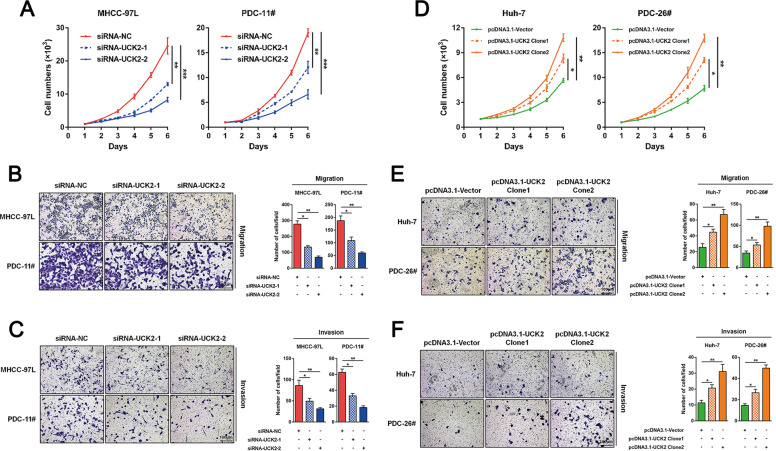
*UCK2* promotes HCC proliferation, migration and invasion in vitro. **a** CCK-8 assays were performed to test the effects of UCK2 knockdown on the proliferation of MHCC-97L and PDC-11# cells in vitro. **b**–**c** Transwell-migration (**b**) and -invasion (**c**) assays were performed to test the effects of UCK2 knockdown on the migration and invasion abilities of MHCC-97L and PDC-11# cells in vitro. **d** CCK-8 assays were performed to test the effects of UCK2 overexpression on the proliferation of Huh-7 and PDC-26# cells in vitro. **e**–**f** Transwell-migration (**e**) and -invasion (**f**) assays were performed to test the effects of UCK2 overexpression on the migration and invasion abilities of Huh-7 and PDC-26# cells in vitro. Scale bar = 100 μm. *, *p* < 0.05; **, *p* < 0.01; ***, *p* < 0.001. Error bars indicate means ± SD.

*UCK2* expression was first examined in 13 cell lines including one normal hepatocyte cell lines (LO2), five commercial available HCC cell lines (Huh-7, HepG2, HCC-LM3, SMMC-7721, and MHCC-97L) and seven home-made primary HCC cell lines (PDC-26#, PDC-14#, PDC-12#, PDC-9#, PDC-23#, PDC-10#, and PDC-11#). Enhanced *UCK2* expression was observed in the 12 HCC cell lines compared with LO2 cells (Fig. S2A). However, *UCK1* did not exhibit such expression differences (Fig. S2B). Furthermore, HCC cells were chosen for loss- or gain-of-function studies due to their high or low endogenous UCK2 levels. *UCK2* knockdown or overexpression was confirmed (Fig. S2C, F). CCK8, colony formation, and Edu incorporation assays showed reduced proliferation in HCC cells with *UCK2* knockdown (Figs. 2A, S2D, E) and enhanced proliferation with *UCK2* overexpression (Figs. 2D, S2G, H). In addition, transwell-migration and -invasion assays showed that *UCK2* knockdown or overexpression respectively impaired or promoted migration and invasion abilities of HCC cells (Fig. 2B-C and E–F). These findings indicate that *UCK2* has an oncogenic role in promoting HCC proliferation, migration, and invasion in vitro.

### UCK2-mediated metastasis enhancement in HCCs is independent on its catalytic activity

To investigate whether the oncogenic role of UCK2 is dependent on its catalytic activity, we replaced UCK2 Asp62, a conserved amino acid residue which was found to be essential for UCK2’s catalytic activity^30,33^, with Ala (UCK2^D62A^), and stable cell lines were respectively established (Fig. S3A). Neither UCK2^WT^ nor UCK2^D62A^ had a significant effect on UCK1 expression (Fig. S3B). The catalytic activity loss of UCK2^D62A^ was examined by cytotoxicity assays by using the cytotoxic pyrimidine analogues 5-Fluorouridine (5-FUrd) and 3′-C-ethynylcytidine (ECyd)^37^. Unlike UCK2^WT^, UCK2^D62A^ failed to sensitize Huh-7 and PDC-26# cells to 5-FUrd and ECyd (Fig. S3C, D), suggesting the deprived catalytic activity in UCK2^D62A^.

**Fig. 3:**
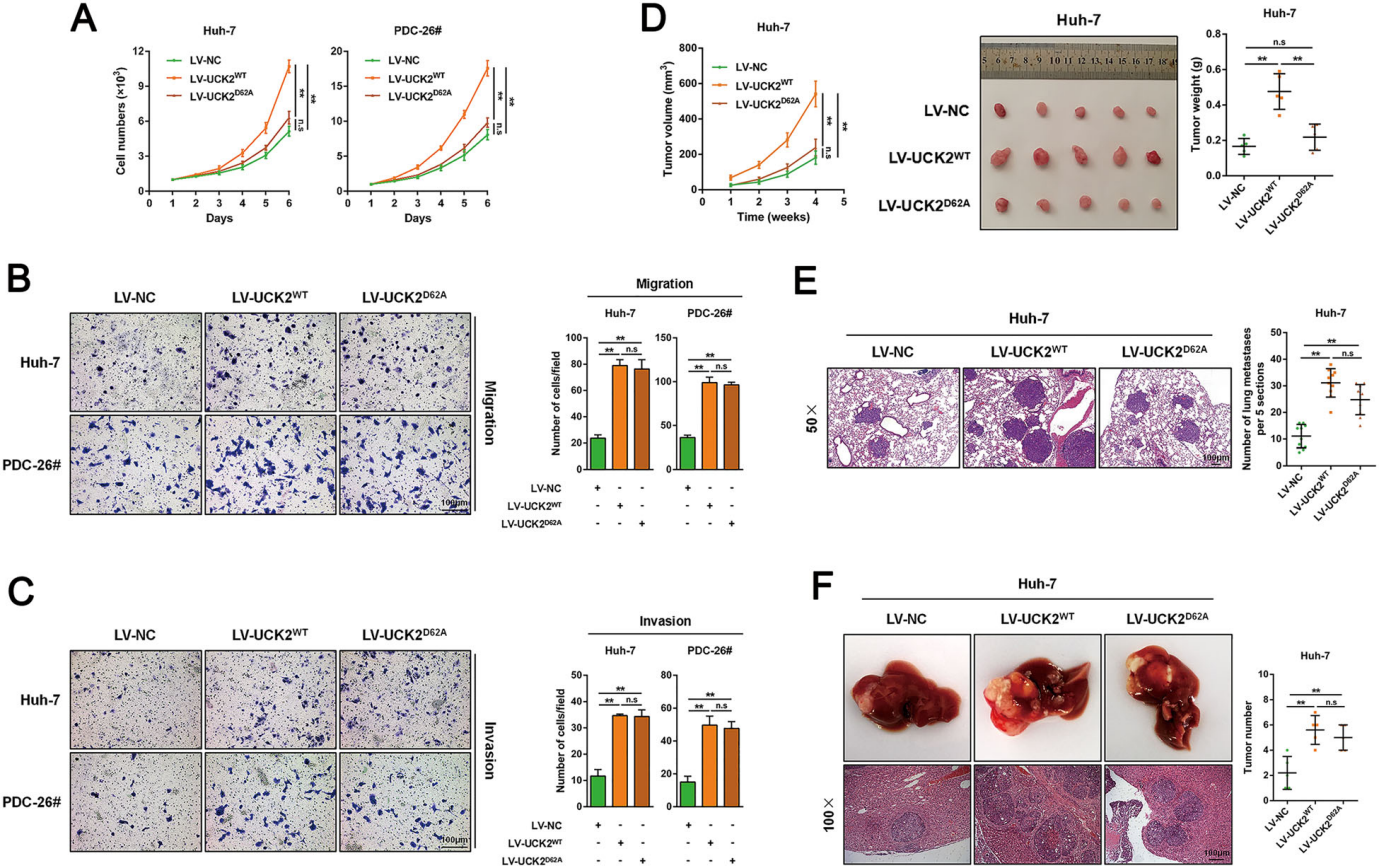
UCK2 non-catalytically promotes metastasis in HCCs. **a** CCK-8 assays were performed to test the effects of UCK2^WT^ or UCK2^D62A^ overexpression on the proliferation of Huh-7 and PDC-26# cells in vitro. **b**–**c** Transwell-migration **(b**) and -invasion **(c**) assays were performed to test the effects of UCK2^WT^ or UCK2^D62A^ overexpression on the migration and invasion abilities of Huh-7 and PDC-26# cells in vitro. **d** Tumor growth curves (left), end-point tumor photography (middle) and tumor weight (right) in Huh-7 cells with either UCK2^WT^ or UCK2^D62A^ overexpression. **e** Representative images (left) and statistic results (right) of lung sections with metastasis foci in Huh-7 cells with either UCK2^WT^ or UCK2^D62A^ overexpression in a pulmonary metastatic model. **f** Representative images (left) and statistic results (right) of intrahepatic metastasis foci in Huh-7 cells with either UCK2^WT^ or UCK2^D62A^ overexpression in a orthotopic implanted intrahepatic metastastic model. Scale bar = 100 μm. **, *p* < 0.01; n.s no significance. Error bars indicate means ± SD.

Correspondingly, CCK8, colony formation and Edu incorporation assays demonstrated that, compared with UCK2^WT^, UCK2^D62A^ lost the growth advantages (Figs. 3A and S3E–F). Interestingly, both UCK2^WT^ and UCK2^D62A^ showed comparable migration and invasion enhancement (Fig. 3B, C), suggesting that UCK2 may non-catalytically promotes HCC metastasis. In line with our in vitro findings, the in vivo xenograft model showed that UCK2 knockdown significantly inhibited tumor growth (Fig. S3G), whereas UCK2^WT^ but not UCK2^D62A^ greatly accelerated tumor growth (Fig. 3D). Meanwhile, the lung metastasis model showed that UCK2 knockdown impaired lung metastasis (Fig. S3H), whereas both UCK2^WT^ and UCK2^D62A^ greatly increased lung metastasis although the size of metastatic foci were smaller in UCK2^D62A^ group (Fig. 3E). Similar results were observed in intrahepatic metastastic models (Figs. 3F and S3I). Therefore, these findings indicate that UCK2-mediated metastasis enhancement is independent on its catalytic activity.

### AKT activation is required for the non-catalytic role of UCK2 in promoting HCC metastasis

**Fig. 4:**
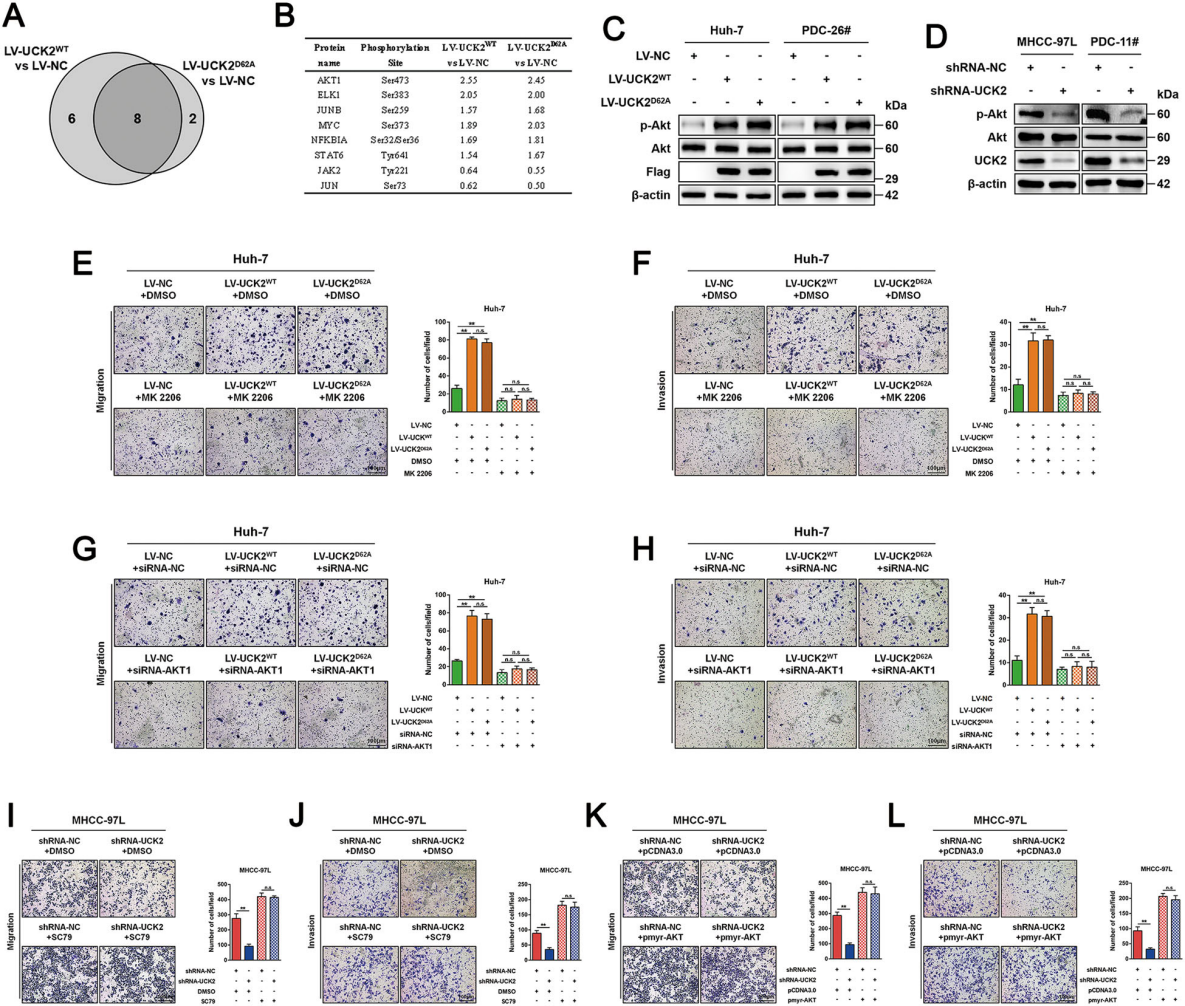
AKT activation is required for the non-catalytic role of UCK2 in promoting HCC metastasis. **a** Eight common phosphorylation changes collected from Huh-7 cells with UCK2^WT^ or UCK2^D62A^ overexpression were presented in a Venn diagram. **b** Details of the eight common phosphorylation signal changes. **c**–**d** Confirmation of UCK2-induced AKT activation by western blot in Huh-7 and PDC-26# cells with UCK2^WT^/UCK2^D62A^ overexpression (**c**) and in MHCC-97L and PDC-11# cells with UCK2 knockdown (**d**). **e**–**f** Transwell-migration (**e**) or -invasion (**f**) assays in Huh-7 cells with UCK2^WT^/UCK2^D62A^ overexpression and MK-2206-mediated AKT inhibition. **g**–**h** Transwell-migration (**g**) or -invasion (**h**) assays in Huh-7 cells with UCK2^WT^/UCK2^D62A^ overexpression and siRNA-mediated AKT downregulation. **i**–**j** Transwell-migration (**i**) or -invasion (**j**) assays in MHCC-97L cells with endogenous UCK2 knockdown and treatment of an AKT1 activator, SC79. **k**–**l** Transwell-migration (**k**) or -invasion (**l**) assays in MHCC-97L cells with endogenous UCK2 knockdown and overexpression of constantly activated AKT1, pmyr-AKT1. Scale bar = 100 μm. **, *p* < 0.01. Error bars indicate means ± SD.

To investigate the mechanism by which UCK2 non-catalytically promotes HCC metastasis, a cancer signaling phospho-antibody array was performed (Fig. S4A). Compared with the vector control, 14 and 10 phospho-candidates with significant phosphorylation intensity (Phos-Ins) alterations (FC ≥ 1.5 or ≤−1.5) were respectively selected from Huh-7 cells with either UCK2^WT^ or UCK2^D62A^ overexpression (Fig. 4A and Table S5). Phosphorylation at the Ser473 of AKT1 was the most elevated one in the eight common altered phospho-sites between UCK2^WT^ and UCK2^D62A^ groups (Fig. 4B and Table S5). Furthermore, increased or decreased phosphorylation at AKT1 on Ser473 was confirmed in HCC cells with UCK2^WT^/UCK2^D62A^ overexpression or UCK2 knockdown, respectively (Fig. 4C, D). To determine whether AKT1 activation was required for UCK2^WT^/UCK2^D62A^-mediated metastasis enhancement. MK-2206 (an AKT inhibitor) (Fig. S4B) or AKT1 knockdown (Fig. S4C) significantly inhibited UCK2^WT^/UCK2^D62A^-induced migration (Fig. 4E, G) and invasion (Fig. 4F, H) in vitro. Moreover, AKT1 activation, by either SC79 (an activator of AKT) (Fig. S4D) or pmyr-AKT (a constitutively active AKT) (Fig. S4E), greatly rescued the inhibition on migration and invasion induced by UCK2 knockdown (Fig. 4I–L). These findings indicate that UCK2 can non-catalytically induce AKT1 activation, which is required for the non-catalytic role of UCK2 in promoting HCC metastasis.

### UCK2 non-catalytically induces AKT activation through enhanced EGFR pathway activity

**Fig. 5:**
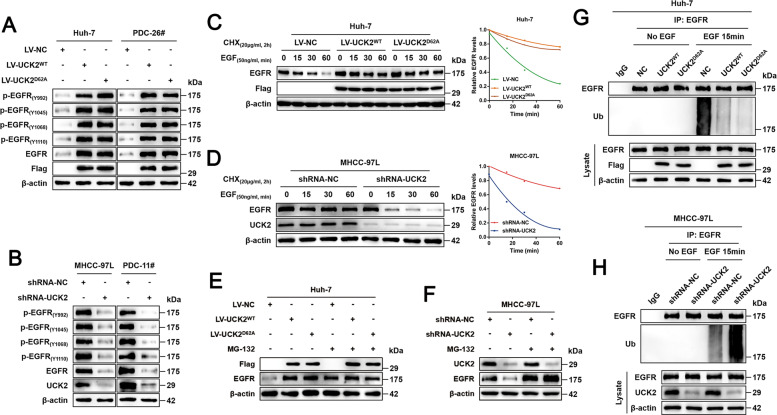
UCK2 non-catalytically activates EGFR-AKT pathway by inhibiting EGF-induced EGFR ubiquitination and degradation. **a**–**b** Confirmation of UCK2 non-catalytically inducing EGFR activation by western blot to probe total and phosphorylated EGFR levels [p-EGFR_(Y992)_, p-EGFR_(Y1045)_, p-EGFR_(Y1068)_ and p-EGFR_(Y1110)_] in Huh-7 and PDC-26# cells with UCK^WT^/UCK2^D62A^ overexpression (**a**) or in MHCC-97L and PDC-11# cells with endogenous UCK2 knockdown (**b**). **c**–**d** Cycloheximide (CHX) chase assays to evaluate the role of UCK2 on EGF-induced EGFR degradation in Huh-7 cells with UCK^WT^/UCK2^D62A^ overexpression (**c**) or in MHCC-97L cells with endogenous UCK2 knockdown (**d**). **e**–**f** Proteasomal degradation was inhibited by MG-132, and EGFR protein levels were determined in Huh-7 cells with UCK^WT^/UCK2^D62A^ overexpression (**e**) or in MHCC-97L cells with endogenous UCK2 knockdown (**f**). **g**–**h** Detection of EGFR ubiquitination levels in Huh-7 cells with UCK2^WT^/UCK2^D62A^ overexpression (**g**) or in MHCC-97L cells with endogenous UCK2 knockdown (**h**) in the context of with or without EGF treatment.

Receptor Tyrosine Kinases (RTKs) are a family of proteins whose activation can activate oncogenic AKT^23^. To further investigate the mechanism by which UCK2 non-catalytically induces AKT1 activation, we performed a second-round scrutiny focusing on phosphorylation changes in RTKs in our phospho-antibody array data. UCK2^WT^/UCK2^D62A^-induced phosphorylation elevation was only observed in EGFR but not in other RTKs such as HER2, IGF1R, VEGFR2 and so on (Table S6). Interestingly, in addition to inducing EGFR phosphorylation, UCK2^WT^/UCK2^D62A^ parallel increased EGFR protein levels, which results in comparable Phos-Ins of EGFR in cells transfected with UCK2^WT^, UCK2^D62A^ or control vectors (Table S6), explaining why we did not find significant Phos-In alterations in EGFR (Fig. 4B and Table S5). In addition, UCK2-mediated EGFR activation was confirmed by western blotting (Fig. 5A, B). Consistent with our array data, UCK2 had no effect on HGF-induced AKT activation but greatly affected EGF-induced AKT activation (Fig. S5A–D). Interestingly, the phosphorylation levels of other downstream targets of EGFR pathway (p-p38, p-ERK1/2 and p-JNK1/2) did not appear to change significantly when UCK2 was overexpressed or interfered (Fig. S5E–F), which was in line with our array data. Moreover, UCK1 loss or overexpression did not affect EGFR signaling, including changes in Akt phosphorylation (Fig. S5G–H). These findings suggest that UCK2 may non-catalytically induce AKT activation through activating EGFR pathway.

### UCK2 non-catalytically stabilizes EGFR proteins by blocking EGF-induced EGFR ubiquitination

Given that UCK2 non-catalytically increases EGFR levels and activates EGFR-AKT pathway, we first examined whether UCK2 regulates EGFR transcriptionally. qRT-PCR assays indicated that either UCK2^WT^/UCK2^D62A^ overexpression or UCK2 knockdown had no effect on EGFR transcription (Fig. S5I, J), suggesting that UCK2 may non-catalytically affect EGFR protein stability. Cycloheximide (CHX) chase assays showed that, in response to EGF treatment, UCK2^WT^/UCK2^D62A^ overexpression or UCK2 knockdown greatly retarded or accelerated EGFR degradation, respectively (Fig. 5C, D). Moreover, these differences disappeared when protein degradation was inhibited by proteasome inhibitor MG-132, confirming the non-catalytical effect of UCK2 on EGFR degradation (Fig. 5E, F). Ubiquitination of EGFR is critical for EGF-induced EGFR degradation via either proteasomal or lysosomal degradation pathways^12,27^, we investigated whether UCK2 non-catalytically affects EGF-induced EGFR ubiquitination. As shown in Fig. 5G, H, UCK2^WT^/UCK2^D62A^ overexpression greatly blocked EGF-induced EGFR ubiquitination whereas UCK2 knockdown significantly enhanced this process. These findings suggest that UCK2 non-catalytically stabilizes EGFR proteins by inhibiting EGF-induced EGFR ubiquitination.

### Physical interaction of UCK2-EGFR is required for the non-catalytic activities of UCK2

**Fig. 6:**
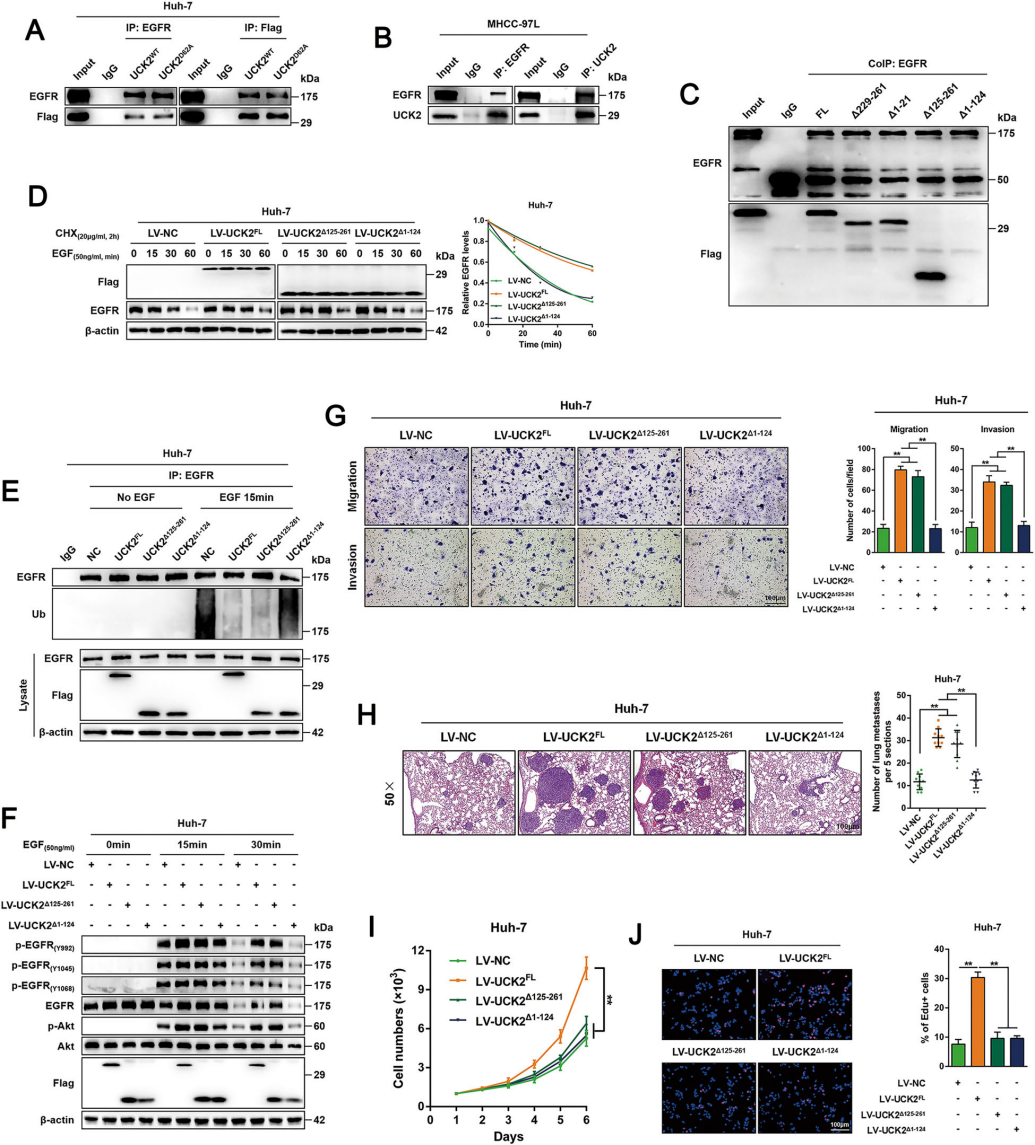
Physical interaction of UCK2-EGFR is required for UCK2-mediated non-catalytic blockage on EGF-induced EGFR ubiquitination and degradation. **a**–**b** Reciprocal IP assays of EGFR and UCK2 in Huh-7 cells with UCK2^WT^/UCK2^D62A^ overexpression (**a**) or in MHCC-97L cells with high endogenous UCK2 expression (**b**). **c** IP assays of EGFR and UCK2 in Huh-7 cells overexpressing indicated UCK2 full length and mutants. **d** Cycloheximide (CHX) chase assays to examine EGFR protein stability in response to EGF in Huh-7 cells overexpressing indicated UCK2 full length and mutants. **e** After 18 h of serum starvation, EGFR ubiquitination levels were detected in Huh-7 cells overexpressing indicated UCK2 full length and mutants. **f** After 18 h of serum starvation, EGF-induced EGFR-AKT activation was detected of in Huh-7 cells overexpressing indicated UCK2 full length and mutants with or without EGF treatment. **g** Transwell-migration and -invasion assays in Huh-7 cells overexpressing indicated UCK2 full length and mutants. **h** Representative images (left half) and statistic results (right half) of lung sections with metastasis foci in Huh-7 cells overexpressing indicated UCK2 full length and mutants in a pulmonary metastatic model. **i**–**j** CCK-8 (**i**) and EdU incorporation (**j**) assays were performed to test the effects of UCK2^FL^, UCK2^Δ125-261^, or UCK2^Δ1-124^ on the proliferation of Huh-7 cells in vitro. Scale bar = 100 μm. **, *p* < 0.01. Error bars indicate means ± SD.

A previous EGFR interactome study has suggested a possible EGFR-UCK2 interaction^34^. Here, physical UCK2-EGFR interaction was confirmed by reciprocal IP in Huh-7 cells with UCK2^WT^/UCK2^D62A^ overexpression and in MHCC-97L cells with high endogenous UCK2 expression (Fig. 6A, B). Such a UCK2-EGFR interaction was exclusively detectable in the presence of EGF (Fig. S6A, B). Moreover, the proximity ligation assay confirmed the endogenous interaction of UCK2 and EGFR in presence of EGF (Fig. S6C). To investigate whether UCK2-EGFR interaction is required for the non-catalytic activities of UCK2, we constructed a series of truncated UCK2 mutants (Fig. S6D, E). Reciprocal IP showed that the mutant without N-terminal half (UCK2^Δ1-124^) failed to interact with EGFR (Figs. 6C and S6F). CHX chase assays showed that UCK2^FL^ or UCK2^Δ125-261^ significantly delayed EGF-induced EGFR degradation, whereas UCK2^Δ1-124^ failed to do so (Fig. 6D). Furthermore, unlike UCK2^FL^ and UCK2^Δ125-261^ which significantly blocked EGF-induced EGFR ubiquitination, UCK2^Δ1-124^ had no effect on this process (Fig. 6E). Correspondingly, EGF treatment greatly enhanced EGFR-AKT activation in Huh-7 cells overexpressing UCK2^FL^ or UCK2^Δ125-261^ but not in cells overexpressing UCK2^Δ1-124^ (Fig. 6F). In line with this notion, compared with UCK2^Δ125-261^, UCK2^Δ1-124^ failed to enhance in vitro migration/invasion and in vivo lung metastasis (Fig. 6G, H), although both UCK2^Δ125-261^ and UCK2^Δ1-124^ showed no effect on HCC proliferation (Fig. 6I, J). Therefore, these findings indicate that the UCK2-EGFR interaction is critical for the non-catalytic roles of UCK2 in HCC.

### Concurrent pharmacological targeting UCK2 and EGFR leads to synergistic suppression of HCC progression

**Fig. 7:**
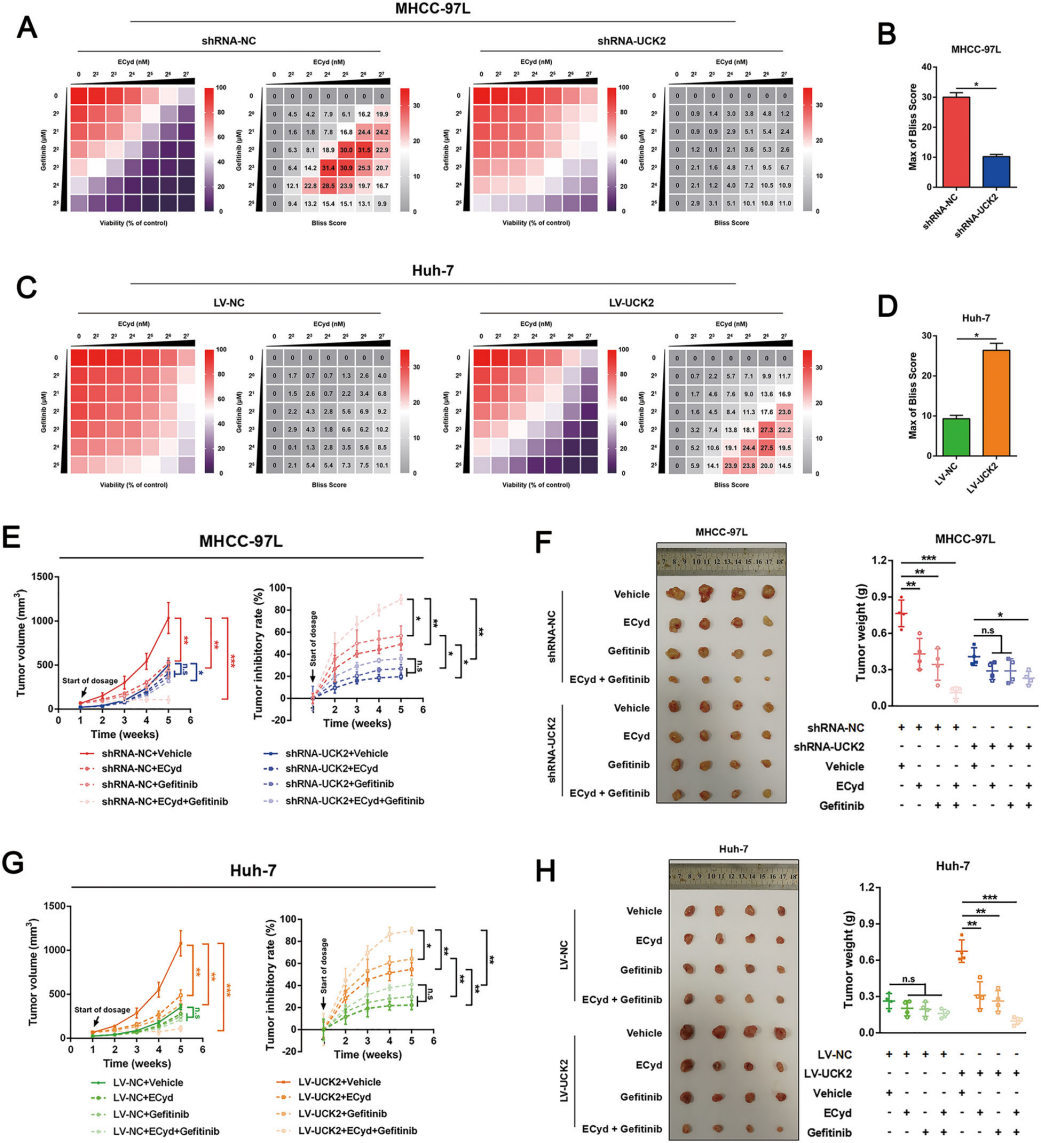
Concurrent pharmacological targeting UCK2 and EGFR leads to synergistic effects on inhibition of HCC growth. **a**–**d** A bliss independent model to evaluate the synergistic cytotoxic effects of concurrent targeting EGFR by Gefitinib and UCK2 by ECyd in MHCC-97L cells with endogenous UCK2 knockdown (**a**–**b**) or in Huh-7 cells with UCK2 overexpression (**c**–**d**). **e**–**h** Tumor volume, tumor inhibitory rate, end-point tumor photography and tumor weight were presented in the xenograft mouse models to evaluate the synergistic effects of concurrent targeting EGFR by Gefitinib and UCK2 by ECyd on the inhibition of tumor growth in MHCC-97L cells with or without endogenous UCK2 knockdown (**e**–**f**) or in Huh-7 cells with or without UCK2 overexpression (**g**–**h**). *, *p* < 0.05; **, *p* < 0.01; ***, *p* < 0.001; n.s no significance. Error bars indicate means ± SD.

Since the metabolic and non-metabolic roles of UCK2, we speculated synergistical inhibition on HCC might be achieved by concurrent pharmacologic targeting UCK2 and EGFR. To test this point, we first examined whether UCK2 would affect the efficacy of cytotoxic pyrimidine analogues (5-Furd or ECyd) and EGFR inhibitors (Erlotinib or Gefitinib) in HCCs. UCK2 knockdown induced significant resistance on cytotoxic pyrimidine analogues (fold change of IC50: 2.88-fold for 5-FUrd and 3.27-fold for ECyd; Fig. S7A) and EGFR inhibitors (fold change of IC50: 2.61-fold for Erlotinib and 2.89-fold for Gefitinib; Fig. S7B), whereas overexpression greatly sensitized to them (fold change of IC50: 0.27-fold for 5-FUrd, 0.31-fold for ECyd, 0.35-fold for Erlotinib and 0.34-fold for Gefitinib; Fig. S7C, D), suggesting the synergistic potential on targeting both UCK2 and EGFR. Then, dose matrix experiments were performed in combination of ECyd and Gefitinib, and synergistic effects were analyzed using the Bliss independent model^41^. Combined treatment of ECyd and Gefitinib was more effective in MHCC-97L (high endogenous UCK2 expression) than in Huh-7 cells (low endogenous UCK2 expression) (Fig. S7E–G). Consistently, UCK2 knockdown abrogated such synergistic effects (Fig. 7A, B), while overexpression enhanced them (Fig. 7C, D). More importantly, the xenograft mouse models further validated the synergistic effects of the combined ECyd and Gefitinib treatment in inhibiting tumor growth in MHCC-97L (Fig. 7E, F), and in Huh-7 cells overexpressing UCK2 (Fig. 7G, H). Body weights of mice had not significantly changed between different treatment groups (Fig. S7H, I). These data indicate that concurrent pharmacological targeting UCK2 and EGFR has synergistic effect on inhibition of HCC progression.

### Preclinical evaluation of the synergistic suppression in patient-derived HCC cells and xenografts

**Fig. 8:**
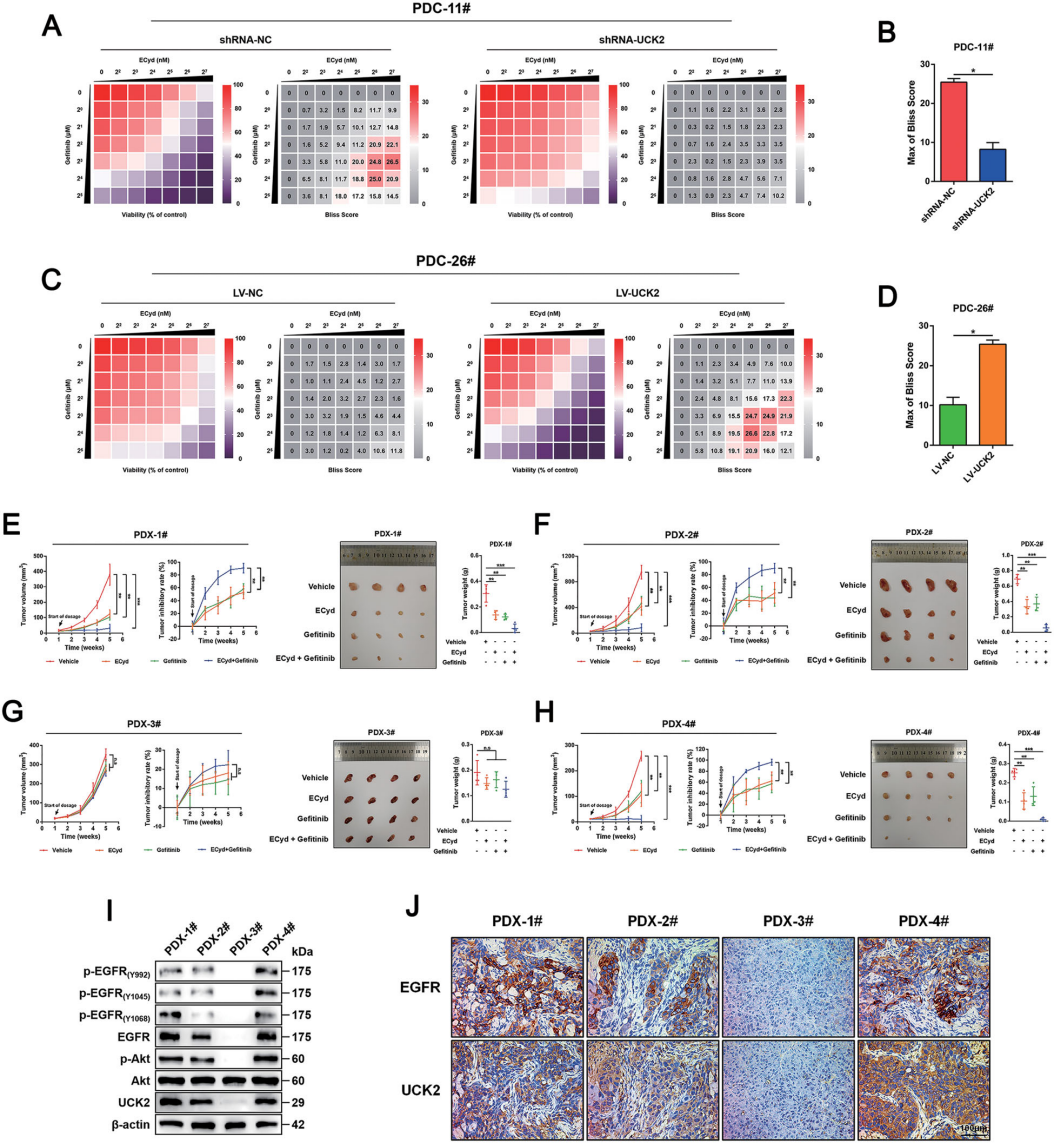
Preclinical evaluation of the synergistic suppression in patient-derived HCC cells and xenografts. **a**–**d** A bliss independent model to evaluate the synergistic cytotoxic effects of concurrent targeting EGFR by Gefitinib and UCK2 by ECyd in PDC-11# cells with endogenous UCK2 knockdown (**a**–**b**) or in PDC-26# cells with UCK2 overexpression (**c**–**d**). **e**–**h** Tumor volume, tumor inhibitory rate, end-point tumor photography and tumor weight were presented in four established PDX HCC tumors (**e**, PDX-1#; **f**, PDX-2#; **g**, PDX-3#; **h**, PDX-4#) in response to treatments of vehicle, ECyd, Gefitinib, or combined ECyd and Gefitinib. **i** Protein levels of EGFR, UCK2 and phosphorylated EGFR and AKT were detected in the four PDX HCC tumors by western blot. **j** Protein levels of EGFR and UCK2 were detected in the four PDX HCC tumors by IHC assays. *, *p* < 0.05; **, *p* < 0.01; ***, *p* < 0.001; n.s no significance. Error bars indicate means ± SD.

Patient-derived cells (PDC) and xenograft (PDX) models were used to predict the clinical responses to such a combined treatment in HCC patients^6,15^. In line with commercial available HCC cells, UCK2 knockdown induced significant resistance on cytotoxic pyrimidine analogues and EGFR inhibitors in PDC-11# cells (Fig. S8A, B), whereas overexpression UCK2 in PDC-26# cells greatly sensitized to them (Fig. S8C, D). Moreover, dose matrix experiments showed that the combined treatment was more effective in PDC-11# (high endogenous UCK2 expression) than in PDC-26# cells (low endogenous UCK2 expression) (Fig. S8E–G). UCK2 knockdown in PDC-11# cells abrogated such synergistic effects (Fig. 8A, B), whereas overexpression enhanced them in PDC-26# cells (Fig. 8C, D). Furthermore, in vivo combined ECyd and Gefitinib treatment showed synergistic effects in 3 out of 4 PDX tumors (Fig. 8E–H). More importantly, high UCK2 and EGFR protein and EGFR activation levels were observed in all the three responsive PDX tumors by western blot (Fig. 8I). Meanwhile, IHC assays confirmed high UCK2 and EGFR protein levels in all the three responsive PDX tumors (Fig. 8J). In addition, either single or combined drug treatment did not cause significant body weight changes (Fig. S8H). These findings preclinically confirmed the pharmacological efficacy and safety of simultaneous targeting the metabolic and non-metabolic functions of UCK2 in HCC.

## Discussion

In present study, we confirm up-regulated UCK2 serves as an independent risk factor of poor prognosis in HCCs. UCK2 plays a pro-oncogenic role by promoting tumor cell proliferation and metastasis. Interestingly, UCK2 catalytically promotes tumor cell proliferation but non-catalytically enhances tumor cell metastasis. Mechanistically, UCK2 can non-catalytically activate EGFR-AKT pathway by interacting with EGFR to inhibit EGF-induced EGFR ubiquitination and degradation, resulting in enhanced HCC metastasis. Pharmaceutically, we demonstrate the synergistic effects in treatment of HCCs by concurrently targeting UCK2 and EGFR in both xenograft and PDX models.

Despite the early observation of aerobic glycolysis by Otto Warburg (the ‘Warburg effect’) in the 1960’s, cancer associated metabolic reprogramming has been long neglected due to simple interpretation of this alteration as a passive response to fulfill the high energy and nutrient demands of tumor cells^39^. Recently, some studies have demonstrated the existence of onco-metabolites to activate oncogenic signalings to potentiate malignant transformation, suggesting metabolism reprogramming as a driven force of cancer^39^. Previous metabolism investigations mainly focus on the role of metabolic reprogramming in promoting tumor cell proliferation^20^, leading to the generation of most metabolism-based anticancer drugs targeting cancer cell proliferation. Recently, the pro-metastasis role of metabolism reprogramming has been revealed. For example, elevated methylglyoxal (MG) levels induced by aerobic glycolysis enhanced the growth and metastasis in breast cancer cells by increasing nuclear accumulation of YAP;^26^ and elevated free fatty acid levels and uptake contribute to epithelial-mesenchymal transition induction in HCC cells by activating Wnt and TGF-β signaling^24^. Given that metastasis is a less frequent phenotype in normal cells compared with proliferation, targeting metastasis may serve as a more selective strategy in developing novel metabolism-based anticancer drugs. In the current study, we demonstrate that UCK2 promotes both proliferation and metastasis in HCCs, suggesting that UCK2 is a potential metabolism-based therapeutic target for HCC treatment.

In the context of metabolism reprogramming, many metabolic enzymes, involved in glycolysis^19^, lipogenesis^31^, amino acid biosynthesis^3^ and nucleotide biosynthesis^35^, are up-regulated to support anabolic growth of cancer cells. To further complicate the matter, some recent studies even demonstrate the non-metabolic roles of some key metabolic enzymes, such as PKM2 and PGK1, in promoting tumor progression^22^. One kind of interesting non-metabolic role comes from the kinase activity of those metabolism enzymes to phosphorylate non-metabolite substrates, such as proteins. For examples, PKM2 has been reported to function as a kinase of multiple proteins including histone H3, STAT3 and SREBPs to promote tumor progression; and PGK1 phosphorylates Beclin1 and pyruvate dehydrogenase kinase isozyme 1 to support tumor development^22^. In the current study, we demonstrate that UCK2 also has a non-metabolic role in promoting HCC development. Interestingly, this non-metabolic role of UCK2 does not require its kinase activity, because kinase-dead UCK2 (UCK2^D62A^) remains functional in promoting HCC metastasis. This finding expands our understanding of the non-metabolic roles performed by metabolic enzymes.

Genetic studies have disclosed the high correlation of genomic EGFR amplification/mutations with EGFR overexpression/activation in many malignancies^9,11,14,32^, but EGFR amplification/mutations are rare in HCCs^8,13^, despite the high EGFR overexpression reported in 60–85% of HCCs^2,16^. We demonstrate that UCK2 interacts with EGFR to impair EGF-induced EGFR ubiquitination, resulting in stabilization of EGFR. Notably, our finding is a bit discrepant with a previous EGFR interactome study showing that such an EGFR-UCK2 interaction was in an EGF-independent manner. Such an EGF-independent EGFR-UCK2 interaction may result from the possible autonomous activation of EGFR due to their overexpression of EGFR in HEK293 cells^5,34^. We demonstrate that the presence of EGF is required for EGFR-UCK2 interaction, suggesting that EGF-induced EGFR modification or internalization may be required for EGFR-UCK2 interaction.

Although pyrimidine biosynthesis has been identified as one of ideal targets for cancer treatment due to its frequent up-regulation in many malignancies^7^, several clinical trials on the inhibitors antagonizing key enzymes of de novo pyrimidine biosynthesis have been failed partly because of the activation and up-regulation of the salvage pyrimidine biosynthesis^21^. For example, compared with normal colon, the activity elevation of UCK (in salvage pathway) was much higher than that of orotidine 5′-phosphate (OMP) decarboxylase (in de novo pathway)^1^. As a rate-limiting enzyme of salvage pyrimidine biosynthesis, UCK2 overexpression has been reported in many types of cancers including HCCs^40^, but the biological function of UCK2 in HCCs is underexplored. In addition to our finding of UCK2-mediated HCC proliferation, one most interesting finding of this study is that we unexpectedly found that UCK2 can non-catalytically promote HCC metastasis. These findings suggest that UCK2 may have divergent pro-tumor functions either dependent or independent on its catalytic activity. This may partially explain the failure of a clinical trial of nucleoside 3′-C-ethynylcytidine (TAS-106), a cytidine analogue subjected to UCK2 phosphorylation, in treatment of head and neck cancer and nasopharyngeal cancer^36^.

In the current study, we demonstrate that UCK2 catalytically promotes HCC proliferation and non-catalytically enhances HCC metastasis by activating the EGFR-AKT pathway, which inspired us to design a combined drug treatment to simultaneously block the catalytic dependent and independent functions of UCK2. Concurrent pharmacological targeting on UCK2 by ECyd and EGFR by Gefitinib showed synergistic effects in treatment of HCCs, suggesting that the non-metabolic roles of targeted metabolic enzymes may need to be considered in metabolism-based therapy for better efficacy.

## Supplementary information

Supplementary Information-Clean revised version ONCSIS-20-0249RRR

Table S1

Table S2

Table S3

Table S4

Table S5

Table S6

Table S7

Figure S1

Figure S2

Figure S3

Figure S4

Figure S5

Figure S6

Figure S7

Figure S8
