# Supplementary material for: Non-metabolic role of UCK2 links EGFR-AKT pathway activation to metastasis enhancement in hepatocellular carcinoma
Source: Oncogenesis. 2020 Dec 4;9(12):103. doi: 10.1038/s41389-020-00287-7 (PMC7718876; doi:10.1038/s41389-020-00287-7)
Supplement: Supplementary file 1 — Supplementary Information-Clean revised version ONCSIS-20-0249RRR [file 41389_2020_287_MOESM1_ESM.doc]

**Supplementary Materials and Methods**

**Plasmids, Stable cell line establishment and RNA interfering**

Human full-length or truncated UCK2 cDNA were inserted to the lentiviral vector pLenti-CMV-MCS-3FLAG-PGK-Puro plasmids by Obio Company (Shanghai, China), and site-directed mutagenesis replacing Asp62 with Ala was further performed in full-length plasmid. Short hairpin RNA (shRNA) targeting UCK2 was constructed into pGLVH1/GFP-Puro plasmids by Genepharma Company (Shanghai, China). To construct stable cell lines with overexpression or downregulation of UCK2, lentivirus vectors were generated based on above plasmids and were designated respectively. Cells were transfected with 2×10^6^ recombinant lentivirus-transducing units and were selected and maintained in the presence of 2 µg/ml puromycin. For RNAi interfering, HCC cells in a 6-well plate were transfected with 100 nM/well siRNA oligonucleotides against UCK2 or AKT1. Transfection steps were following the manufacture’s protocols, using Lipofectamine RNAiMAX (Invitrogen) for the transfection of siRNA oligonucleotides and NEOFECT (NEOFECT Gene Delivery Biosystems) for plasmid transfection. The sequences of siRNAs or shRNAs used were: UCK2-1, 5’-CCCUGGACCUGUAAAGAAA-3’; UCK2-2, 5’-GCCUCAAGAACCUUCUAAU-3’; and AKT1, 5’-GCCUCAAGAACCUUCUAAU-3’.

**Reagents and antibodies**

Reagents used in the experiments: EGF (100-15), HGF (100-39) purchased from Peprotech (USA); MK 2206 (HY-10358), SC79 (HY-18749), Cycloheximide (HY-12320), MG-132 (HY-13259), Gefitinib (HY-50895), Erlotinib (HY-50896), ECyd (HY-16200) purchased from MedChemExpress (USA); 5-FUrd (F5130) purchased from Sigma-Aldrich (USA). Primary antibodies used in western blot and IHC were as follows: UCK1 (12271-1-AP), UCK2 (10511-1-AP), β-actin (20536-1-AP), LMNB1 (12987-1-AP), TUBA1B (11224-1-AP), ATP1A1 (14418-1-AP), ubiquitin (10201-2-AP) purchased from Proteintech Company (Wuhan, China); Flag (14793), AKT (4691), p-AKT (4060), EGFR (2232), p-EGFR-Y992 (2235), p-EGFR-Y1045 (2237), pEGFR-Y1068 (3777) purchased from Cell Signaling Technology Company (USA); and pEGFR-Y1110 (TA325436) purchased from OriGene Technology Company (USA).

**Real-time quantitative PCR analysis**

Total RNA was isolated using the total RNA isolation kit (BioTeke Corporation, Beijing, China), and reversely transcribed with HiScript Q RT SuperMix for qPCR kit (Vazyme Biotech, Nanjing, China). Real-time quantitative PCR was performed using the SYBR Green PCR master mix (Vazyme Biotech) on the CFX96 TouchTM Real-Time PCR Detection System (BIO-RAD, USA). Analysis of relative gene expression was performed in ΔΔCT by comparing the gene of interest CT value to housekeeping gene 18S CT value. Primer sequences were listed in Table S7.

**Western blot analysis**

Total protein was extracted from cells or snap-frozen tissues with RIPA Lysis Buffer (Pierce Biotechnology) and the concentration was measured using a BCA Protein Assay Kit (Pierce Biotechnology). Western blot was performed using specific primary antibodies, followed with a horseradish peroxidase (HPR)-conjugated secondary antibody and then detected using Amersham lmager 600 (General Electric Company, USA).

**Immunohistochemistry and Tissue Microarrays Analysis**

Paraffin-embedded primary tumors were sliced into 6-μm thickness. Then, paraffin-embedded sections were deparaffinized, rehydrated and incubated with 3% hydrogen peroxide for 30 min. Antigen retrieval was done by incubating the sections in citrate buffer (pH 6.0) by boiling for 10 min in the microwave oven. Then, the slides were incubated with specific primary antibodies overnight at 4°C. Following 60 min incubation with HPR-conjugated secondary antibody, sections were developed in DAB solution (KIT-5004, MXB biotechnologies, Fuzhou, China) under microscopic observation and counterstained with hematoxylin. Photographs of representative fields were captured using the Leica QWin Plus v3 software.

Two tissue microarrays with definite HCC specimens were constructed in this study. All HCC samples in the two TMA cohorts were reviewed histologically by hematoxylin and eosin staining. Representative areas were punched out of the paraffin block and mounted onto a recipient block with a semi-automated TMArrayer (Pathology Devices, MD, USA). Immunohistochemistry assays on tissue microarray were performed as described above. Photographs of representative fields were captured using the Leica QWin Plus v3 software. The Image-Pro Plus v6.0 software (Media Cybernetics Inc, MD, USA) was used to count the integrated optical density (IOD) of each photograph, and the ratio of IOD to total tissue area (AREA) of each photograph was calculated as staining intensity.

**CCK-8, colony formation, and EdU incorporation assays**

For cell viability assays, cells were seeded into 96-well plates at 2 × 10^3^ cells per well and cultured at 37°C. 10% (v/v) of CCK-8 (Dojindo, Kumamoto, Japan) was added to the culture medium and incubation for another 3 hours. Cell viability was monitored by measuring absorbance at 450nm using a SpectraMax i3 microplate reader (Molecular Devices, USA). The experiment was performed in quadruplicate and repeated three times.

Colony formation assays were performed to evaluate long-term proliferation ability in vitro. Cells were seeded into 60 mm dishes at a density of 3×10^3^ cells per dish. After 2 weeks of growth, surviving colonies were fixed and stained with 0.1% crystal violet in 4% paraformaldehyde solution.

EdU incorporation assays were further performed to validate cell viability using the EdU kit (C10310-1, RiboBio, Guangzhou, China). Approximately 1×10^5^ cells were planted in 24-well plates with coverslips and were allowed to adhere. Then, 50μM EdU labeling medium was added to each well and incubated cells for additional 2h at 37°C. The cells were fixed using 4% paraformaldehyde for 30 min and treated with 0.5% Triton X-100 for 10 min at room temperature. Subsequently, the cells were stained with 1 × Apollo® 567 for 20 min and the nuclei of cells were stained with Hoechst 33342. Proliferation index was then determined by quantifying the percentage of EdU-positive cells with fluorescence microscope. All experiments were performed in three biological replicates.

**Transwell-migration and -invasion assays**

For migration and invasion assays, trans-well filter champers (pore size: 8μm; Falcon, USA) and BioCoat matrigel invasion chambers (pore size: 8μm; BD Biosciences, USA) were used according to manufacturer's instructions. Briefly, 5×10^4^ cells were seeded into the upper chamber and allowed to migrate into the lower chamber for 18-24 hours. Cells in the upper chamber were carefully removed using cotton buds and cells at the bottom of the membrane were fixed, stained with crystal violet, and counted under a microscope. Photographs of six randomly selected fields of the fixed cells were captured. All experiments were repeated independently three times. The numbers of migrated cells were counted and expressed as mean ± SD.

**In vivo tumor growth and metastasis assays**

Animal experimental protocols were approved by the Institutional Animal Care and Use Committee of Renji Hospital, School of Medicine, Shanghai Jiao Tong University. Male athymic BALB/c nude mice (4-5 weeks old) were housed and received humane care throughout the experiments. Animal studies were conducted by simple randomization and open-label evaluation.

For subcutaneous xenograft study, 5×10^6^ stale cells were subcutaneously implanted into the right armpit of BALB/C nude mice (n = 5 per group). Tumor sizes were weekly measured with a caliper, and the tumor volume was calculated using the following equation: tumor size = larger diameter × (smaller diameter)^2^ / 2. All mice were sacrificed four weeks later, and the tumors were collected for further study (photographing, weighing, and fixing).

The sections of subcutaneous tumour tissues were futher orthotopic implanted into the liver of nude mice to establish intrahepatic metastastic models. After 8 weeks, mice were sacrificed, and their livers were dissected and examined for tumor numbers and size.

About 5×10^5^ stable cells were injected into the tail vein of 5-week-old male BALB/C nude mice for the establishment of pulmonary metastatic model. All the mice were sacrificed at 10 weeks post injection. The lungs of each mouse were removed and fixed for H&E staining. The average number of lung metastatic foci in each group was examined with a microscopic count assay.

**Co-immunoprecipitation**

Whole cell extracts were prepared in the IP lysis buffer (87787, Thermo Fisher, MA, USA) supplemented with protease inhibitor cocktail (539136, Merck Millipore, Darmstadt, Germany) and centrifuged at 12,000g for 10 min. The supernatants were then incubated with indicated antibodies for 18 hours at 4°C, followed by the addition of Protein A/G Plus-Agarose (20423, Thermo Fisher, MA, USA) for another 2 hours. Total and binding proteins were detected by western blot. Primary antibodies used in this study: UCK2 (10511-1-AP, Proteintech, Wuhan, China), EGFR (2232, Cell Signaling Technology, MA, USA), Flag (14793, Cell Signaling Technology, MA, USA). Normal rabbit IgG (8726, Cell Signaling Technology, MA, USA) was used as controls.

**Dose-response IC50**

Approximately 5 × 10^3^ cells were seeded per well into a 96-well plate 24 h prior to drug treatment. Drugs were threefold diluted in DMSO and kept at 1% (v/v) across all drug concentrations and control. Each drug concentration was tested in triplicate. The viability of cells was assayed using CCK-8. The luminescence signals were detected at 450nm using a SpectraMax i3 microplate reader (Molecular Devices, CA, USA). The relative luminescence units from treated wells were normalized against DMSO control wells and expressed as percentage cell viability. IC50 values were calculated using GraphPad Prism software.

***In Vivo* Drug Studies**

After 1 week of implantation, mice were randomized into indicated groups (n = 4) and subjected to the treatments with vehicle, or Gefitinib (100 mg/kg/d, orally), or ECyd (1 mg/kg/week, intravenously), or combination with Gefitinib (100 mg/kg/d, orally) and ECyd (1 mg/kg/week, intravenously). Weight of mice and tumor volume were measured weekly. Tumor volume = larger diameter × (smaller diameter)^2^ / 2. The inhibitory rate of treated tumors at each time point was also calculated. After 5 weeks, mice were sacrificed and tumors were photographed.

**Figure legends**

**Figure S1. Identification of UCK2 as a key up-regulated metabolic gene in HCCs, related to Figure 1**

1. Volcano plots of differentially expressed genes in GSE14520 (p-value < 0.05, fold change > 1.5 or < -1.5, blue: down-regulated genes, red: up-regulated genes, gray: non-differential genes). (B) Volcano plots of metabolic genes in GSE14520 (yellow: metabolic genes, gray: non-metabolic genes). (C) Volcano plots of differentially expressed metabolic genes in GSE14520 (p-value < 0.05, fold change > 1.5 or < -1.5, green: down-regulated metabolic genes, orange: up-regulated metabolic genes, gray: non-differential or non-metabolic genes). (D-F) Forest plot of the top-10 up-regulated metabolic genes in GSE14520 associated with overall survival (OS) (D), recurrence-free survival (RFS) (E) and early-RFS (within 2 years) (F) in HCC patients. (G) Representative images of immunohistochemistry (IHC) of UCK2 on tissue microarrays. (H-J) Kaplan-Meier analyses of OS, RFS and early-RFS of HCC patients in correlation with high or low UCK2 levels in HCC patients at TNM stage I (H), II (I), or III (J) in TMA Cohort 1 and 2. The absolute number of patients at risk is listed below each curve. Scale bar = 100 µm.

**Figure S2. UCK2 promotes proliferation, migration and invasion in HCC cells, related to Figure 2.**

1. qRT-PCR and western blot analyses of *UCK2* expression levels in 13 cell lines including one normal hepatocyte cell line (LO2), 5 commercial available HCC cell lines (Huh-7, HepG2, HCC-LM3, SMMC-7721 and MHCC-97L) and 7 home-made primary HCC cell lines (PDC-26#, PDC-14#, PDC-12#, PDC-9#, PDC-23#, PDC-10# and PDC-11#). (B) Western blot analysis of *UCK1* expression levels in 13 cell lines. (C) Validation of *UCK2* knockdown in HCC cell lines by qRT-PCR and western blot. (D-E) Colony formation (D) and EdU incorporation (E) assays were performed to test the effects of UCK2 knockdown on the proliferation of MHCC-97L and PDC-11# cells *in vitro*. (F) Validation of *UCK2* overexpression in HCC cell lines by qRT-PCR and western blot. (G-H) Colony formation (G) and EdU incorporation (H) assays were performed to test the effects of UCK2 overexpression on the proliferation of Huh-7 and PDC-26# cells *in vitro*. Scale bar = 100 µm. *, p < 0.05; **, p < 0.01; ***, p < 0.001. Error bars indicate means ± SD.

**Figure S3. The catalytic activity is not required for UCK2-mediated metastasis enhancement in HCCs, related to Figure 3.**

1. Validation of UCK2^WT^ or UCK2^D62A^ overexpression in Huh-7 and PDC-26# cells by western blot. (B) Protein levels of UCK1 in Huh-7 cells with UCK2^WT^ or UCK2^D62A^ overexpression. (C-D) Validation of the impairment of catalytic activity of UCK2^D62A^ by cellular cytotoxicity assays with the treatment of 5-FUrd (C) or ECyd (D). (E-F) Colony formation (E) and EdU incorporation (F) assays were performed to test the effects of UCK2^WT^ or UCK2^D62A^ overexpression on the proliferation of Huh-7 and PDC-26# cells *in vitro*. (G) Tumor growth curves (left), end-point tumor photography (middle) and tumor weight (right) in MHCC-97L cells with UCK2 knockdown. (H) Representative images (left) and statistic results (right) of lung sections with metastasis foci in MHCC-97L cells with UCK2 knockdown in a pulmonary metastatic model. (I) Representative images (left) and statistic results (right) of intrahepatic metastasis foci in MHCC-97L cells with UCK2 knockdown in a orthotopic implanted intrahepatic metastastic model. Scale bar = 100 µm. *, p < 0.05; **, p < 0.01; n.s, no significance. Error bars indicate means ± SD.

**Figure S4. UCK2 can non-catalytically activate AKT, related to Figure 4.**

1. Images of protein array data of Cancer Signaling Phospho-Antibody Array (PCS248; Full Moon Biosystems, CA, USA) in Huh-7 cells transfected with control vector, UCK2^WT^ or UCK2^D62A^ overexpression vectors. (B-C) The effects of MK-2206 (B) or siRNA-AKT1 (C) on the levels of total AKT and phosphorylated (p-AKT) were examined by western blot in Huh-7 cells transfected with control vector, UCK2^WT^ or UCK2^D62A^ overexpression vectors. (D-E) The effects of SC79 (D) or pmyr-AKT (E) on the levels of total AKT and phosphorylated AKT (p-AKT) were examined by western blot in MHCC-97L cells with endogenous UCK2 knockdown.

**Figure S5. UCK2 non-catalytically activates AKT through the EGFR pathway, related to Figure 5.**

(A-B) After 18hrs of serum starvation, HGF induced AKT activation was examined in Huh-7 cells with either UCK2^WT^ or UCK2^D62A^ overexpression (A), or in MHCC-97L cells with endogenous UCK2 knockdown (B) by western blot. (C-D) After 18hrs of serum starvation, EGF induced AKT activation was examined in Huh-7 cells with either UCK2^WT^ or UCK2^D62A^ overexpression (C), or in MHCC-97L cells with endogenous UCK2 knockdown (D) by western blot. (E-F) Phosphorylation levels of p38, ERK1/2 and JNK1/2 were examined in HCC cells with either UCK2^WT^ or UCK2^D62A^ overexpression (E), or endogenous UCK2 knockdown (F) by western blot. (G-H) Phosphorylation levels of Akt, p38, ERK1/2 and JNK1/2 were examined in HCC cells with UCK1 overexpression (G) or knockdown (H) by western blot. (I-J) qRT-PCR analyses of *UCK2* and *EGFR* mRNA levels in Huh-7 cells with either UCK2^WT^ or UCK2^D62A^ overexpression (I), or in MHCC-97L cells with endogenous UCK2 knockdown (J).

**Figure S6. The N-terminal half of UCK2 is required for UCK2-EGFR interaction, related to Figure 6.**

(A-B) After 18hrs of serum starvation, reciprocal IP assays of EGFR and UCK2 were performed in Huh-7 cells with UCK2^WT^/UCK2^D62A^ overexpression (A) or in MHCC-97L cells with high endogenous UCK2 expression (B) in the context of with or without EGF treatment. (C) The endogenous interaction of EGFR and UCK2 in MHCC-97L cells was confirmed by proximity ligation assays in presence and absence of EGF. (D) A schematic of full-length UCK2 (FL) and its deletion mutants (Δ229-261, Δ1-21, Δ125-261 and Δ1-124). (E) Validation of the overexpression of UCK2 full-length (FL) and indicated truncation forms (Δ229-261, Δ1-21, Δ125-261 and Δ1-124) in Huh-7 cells by western blot. (F) Co-immunoprecipitation assays to detect UCK2-EGFR in Huh-7 cells overexpressing UCK2 full-length (FL) and indicated truncation forms (Δ229-261, Δ1-21, Δ125-261 and Δ1-124).

**Figure S7. Synergistic effects of concurrent targeting UCK2 and EGFR in HCC treatment, related to Figure 7.**

(A-B) Cytotoxicity assays of cytotoxic pyrimidine analogues (A) and EGFR inhibitors (B) in MHCC-97L cells with endogenous UCK2 knockdown. (C-D) Cytotoxicity assays of cytotoxic pyrimidine analogues (C) and EGFR inhibitors (D) in Huh-7 cells with UCK2 overexpression. (E-G) Bliss independent model to determine the synergistic effects of combined ECyd and Gefitinib treatment in indicated HCC cell lines. (H-I) The body weight of each mouse was monitored in the xenografts with either single or combined drug treatment. Scale bar = 100 μm. *, p < 0.05; n.s, no significance. Error bars indicate means ± SD.

**Figure S8. Preclinical evaluation of the synergistic suppression in patient-derived HCC cells and xenografts, related to Figure 8.**

1. B) Cytotoxicity assays of cytotoxic pyrimidine analogues (A) and EGFR inhibitors (B) in PDC-11# cells with endogenous UCK2 knockdown. (C-D) Cytotoxicity assays of cytotoxic pyrimidine analogues (C) and EGFR inhibitors (D) in PDC-26# cells with UCK2 overexpression. (E-G) Bliss independent model to determine the synergistic effects of combined ECyd and Gefitinib treatment in indicated HCC cell lines. (H) The body weight of each mouse was monitored in the PDX models with either single or combined drug treatment. Scale bar = 100 μm. *, p < 0.05; n.s, no significance. Error bars indicate means ± SD.
