# Supplementary material for: Non-metabolic role of UCK2 links EGFR-AKT pathway activation to metastasis enhancement in hepatocellular carcinoma
Source: Oncogenesis. 2020 Dec 4;9(12):103. doi: 10.1038/s41389-020-00287-7 (PMC7718876; doi:10.1038/s41389-020-00287-7)
Supplement: Supplementary file 2 — Table S1 [file 41389_2020_287_MOESM2_ESM.doc]

Supplementary Table 1. Clinical Characteristics of the HCC Patients in the Different Cohorts at the Time of Surgery. Related to Figure 1

| **Characteristics** | **TMA Cohort 1** | **TMA Cohort2** | **p-value** |
| --- | --- | --- | --- |
|  | **(n=153)** | **(n=307)** |  |
| **Gender (female/male)** | 25/128 | 32/275 | .070 |
| **Age (years, <50/≥50)** | 83/70 | 154/153 | .409 |
| **AFP (μg/L, <20/≥20)** | 46/107 | 108/199 | .274 |
| **Cirrhosis (no/yes)** | 40/113 | 101/206 | .139 |
| **Tumor size (cm, <5/≥5)** | 67/86 | 126/181 | .574 |
| **Histological grade (I-II/III-IV)** | 35/118 | 75/232 | .713 |
| **Pathological satellite (no/yes)** | 99/54 | 212/95 | .348 |
| **Microvascular Invasion (no/yes)** | 96/57 | 204/103 | .432 |
| **HBsAg (negative/positive)** | 37/116 | 88/219 | .309 |
| **TNM stage (I/II+III)** | 43/110 | 96/211 | .486 |

AFP, alpha-fetoprotein; TNM, tumor-node-metastasis. P < 0.05 was considered statistically significant by χ^2^ test.
