# Supplementary material for: Non-metabolic role of UCK2 links EGFR-AKT pathway activation to metastasis enhancement in hepatocellular carcinoma
Source: Oncogenesis. 2020 Dec 4;9(12):103. doi: 10.1038/s41389-020-00287-7 (PMC7718876; doi:10.1038/s41389-020-00287-7)
Supplement: Supplementary file 3 — Table S2 [file 41389_2020_287_MOESM3_ESM.doc]

**Supplementary Table 2. Association between UCK2 expression and clinicopathologic characteristics of HCC**

patients in TMA cohort 1 and TMA cohort 2. Related to Figure 1

| **Characteristics** | **TMA Cohort 1** | | **p-value** | **TMA Cohort 2** | | **p-value** |
| --- | --- | --- | --- | --- | --- | --- |
|  | **Low (%)**  **(n=76)** | **High (%)**  **(n=77)** |  | **Low (%)**  **(n=153)** | **High (%)**  **(n=154)** |  |
| **Gender**  Female  Male | 10 (13.2)  66 (86.8) | 15 (19.5)  62 (80.5) | .290 | 15 (9.8)  138 (90.2) | 17 (11.0)  137 (89.0) | .723 |
| **Age (years)**  <50  ≥50 | 41 (53.9)  35 (46.1) | 42 (54.5)  35 (45.5) | .941 | 81 (52.9)  72 (47.1) | 73 (47.4)  81 (52.6) | .332 |
| **AFP (μg/L)**  <20  ≥20 | 30 (39.5)  46 (60.5) | 16 (20.8)  61 (79.2) | **.012** | 63 (41.2)  90 (58.8) | 45 (29.2)  109 (70.8) | **.028** |
| **Cirrhosis**  No  Yes | 18 (23.7)  58 (76.3) | 22 (28.6)  55 (71.4) | .492 | 47 (30.7)  106 (69.3) | 54 (35.1)  100 (64.9) | .418 |
| **Tumor size (cm)**  <5  ≥5 | 42 (55.3)  34 (44.7) | 25 (32.5)  52 (67.5) | **.004** | 79 (51.6)  74 (48.4) | 47 (30.5)  107 (69.5) | **<.001** |
| **Histological grade**  I-II  III-IV | 24 (31.6)  52 (68.4) | 11 (14.3)  66 (85.7) | **.011** | 48 (31.4)  105 (68.6) | 27 (17.5)  127 (82.5) | **.005** |
| **Pathological satellite**  No  Yes | 57 (75.0)  19 (25.0) | 42 (54.5)  35 (45.5) | **.008** | 117 (76.5)  36 (23.5) | 95 (61.7)  59 (38.3) | **.005** |
| **Microvascular Invasion**  No  Yes | 58 (76.3)  18 (23.7) | 38 (49.4)  39 (50.6) | **.001** | 117 (76.5)  36 (23.5) | 87 (56.5)  67 (43.5) | **<.001** |
| **HBsAg**  Negative  Positive | 16 (21.1)  60 (78.9) | 21 (27.3)  56 (72.7) | .369 | 40 (26.1)  113 (73.9) | 48 (31.2)  106 (68.8) | .330 |
| **TNM stage**  I  II+III | 29 (38.2)  47 (61.8) | 14 (18.2)  63 (81.8) | **.006** | 62 (40.5)  91 (59.5) | 34 (22.1)  120 (77.9) | **<.001** |

AFP, alpha-fetoprotein; TNM, tumor-node-metastasis. *P* < 0.05 was considered statistically significant by Pearson

χ2 test or Fisher exact test.
