# Supplementary material for: Non-metabolic role of UCK2 links EGFR-AKT pathway activation to metastasis enhancement in hepatocellular carcinoma
Source: Oncogenesis. 2020 Dec 4;9(12):103. doi: 10.1038/s41389-020-00287-7 (PMC7718876; doi:10.1038/s41389-020-00287-7)
Supplement: Supplementary file 4 — Table S3 [file 41389_2020_287_MOESM4_ESM.doc]

Supplementary Table 3. Univariate and multivariate analyses of factors associated with prognosis of HCC patients in TMA cohort 1. Related to Figure 1

| **Variables** | **Categories** | **Overall survival** | | | **Recurrence-free survival** | | | | **Recurrence-free survival _(2 years)_** | | | | | |
| --- | --- | --- | --- | --- | --- | --- | --- | --- | --- | --- | --- | --- | --- | --- |
|  |  | **HR** | **95% CI** | **p-value** | **HR** | **95% CI** | **p-value** | | **HR** | | **95% CI** | | **p-value** | |
| **Univariate analysis** | | | | | | | |  | |  | |  | |  |
| Gender | Male / female | 1.934 | 0.882-4.237 | .100 | 1.439 | 0.760-2.725 | .264 | | 1.383 | | 0.657-2.907 | | .393 | |
| Age (years) | ≥50 / <50 | 0.988 | 0.606-1.610 | .960 | 0.816 | 0.527-1.266 | .365 | | 0.781 | | 0.474-1.285 | | .330 | |
| AFP (μg/L) | ≥20 / <20 | 1.786 | 1.014-3.147 | **.045** | 1.813 | 1.082-3.039 | **.024** | | 2.179 | | 1.160-4.095 | | **.016** | |
| Cirrhosis | Yes / no | 1.048 | 0.602-1.825 | .867 | 1.381 | 0.860-2.217 | .181 | | 1.453 | | 0.859-2.463 | | .688 | |
| Tumor size (cm) | ≥5 / <5 | 2.105 | 1.270-3.489 | **.004** | 2.342 | 1.467-3.739 | **<.001** | | 2.416 | | 1.404-4.157 | | **.001** | |
| Histological grade | III-IV / I-II | 1.485 | 0.807-2.733 | .203 | 1.054 | 0.630-1.765 | .841 | | 1.008 | | 0.563-1.802 | | .979 | |
| Pathological satellite | Yes / no | 1.635 | 0.984-2.717 | .058 | 2.656 | 1.697-4.159 | **<.001** | | 3.040 | | 1.834-5.040 | | **<.001** | |
| Microvascular Invasion | Yes / no | 2.430 | 1.480-3.991 | **<.001** | 3.473 | 2.217-5.443 | **<.001** | | 3.665 | | 2.200-6.108 | | **<.001** | |
| HBsAg | Positive / negative | 1.325 | 0.721-2.435 | .364 | 1.016 | 0.612-1.686 | .950 | | 1.205 | | 0.689-2.105 | | .514 | |
| TNM stage | II+III / I | 1.765 | 1.012-3.079 | **.045** | 4.734 | 2.429-9.229 | **<.001** | | 5.411 | | 2.325-12.596 | | **<.001** | |
| Protein level of UCK2 | High / low | 2.447 | 1.474-4.061 | **.001** | 2.550 | 1.610-4.038 | **<.001** | | 2.553 | | 1.506-4.328 | | **.001** | |
| **Multivariate analysis** | | | | | | | |  | |  | |  | |  |
| AFP (μg/L) | ≥20 / <20 | 1.434 | 0.793-2.596 | .233 | 1.397 | 0.820-2.379 | .218 | | 1.626 | | 0.853-3.098 | | .140 | |
| Tumor size (cm) | ≥5 / <5 | 2.592 | 1.187-5.659 | **.017** | 1.166 | 0.669-2.029 | .588 | | 1.170 | | 0.625-2.190 | | .623 | |
| Pathological satellite | Yes / no | - | - | - | 1.726 | 1.063-2.801 | **.027** | | 1.860 | | 1.076-3.215 | | **.026** | |
| Microvascular Invasion | Yes / no | 2.476 | 1.367-4.485 | **.003** | 2.069 | 1.250-3.426 | **.005** | | 2.152 | | 1.208-3.836 | | **.009** | |
| TNM stage | II+III / I | 2.092 | 0.799-5.495 | .133 | 2.119 | 0.873-5.139 | .097 | | 2.137 | | 0.723-6.322 | | .170 | |
| Protein level of UCK2 | High / low | 1.904 | 1.107-3.276 | **.020** | 1.718 | 1.047-2.821 | **.032** | | 1.547 | | 0.865-2.764 | | .141 | |

AFP, alpha-fetoprotein; TNM, tumor-node-metastasis; HR, hazard ratio; 95% CI, 95% confidence interval. A value of *P* < 0.05 was considered to be significant.
