# Supplementary material for: Non-metabolic role of UCK2 links EGFR-AKT pathway activation to metastasis enhancement in hepatocellular carcinoma
Source: Oncogenesis. 2020 Dec 4;9(12):103. doi: 10.1038/s41389-020-00287-7 (PMC7718876; doi:10.1038/s41389-020-00287-7)
Supplement: Supplementary file 5 — Table S4 [file 41389_2020_287_MOESM5_ESM.doc]

Supplementary Table 4. Univariate and multivariate analyses of factors associated with prognosis of HCC patients in TMA cohort 2. Related to Figure 1

| **Variables** | **Categories** | **Overall survival** | | | **Recurrence-free survival** | | | | **Recurrence-free survival _(2 years)_** | | | | | |
| --- | --- | --- | --- | --- | --- | --- | --- | --- | --- | --- | --- | --- | --- | --- |
|  |  | **HR** | **95% CI** | **p-value** | **HR** | **95% CI** | **p-value** | | **HR** | | **95% CI** | | **p-value** | |
| **Univariate analysis** | | | | | | | |  | |  | |  | |  |
| Gender | Male / female | 1.153 | 0.677-1.965 | .600 | 1.003 | 0.622-1.616 | .991 | | 1.050 | | 0.578-1.908 | | .872 | |
| Age (years) | ≥50 / <50 | 0.976 | 0.710-1.342 | .882 | 0.977 | 0.723-1.318 | .875 | | 0.826 | | 0.572-1.192 | | .307 | |
| AFP (μg/L) | ≥20 / <20 | 1.712 | 1.196-2.451 | **.003** | 1.569 | 1.130-2.179 | **.007** | | 1.571 | | 1.044-2.362 | | **.030** | |
| Cirrhosis | Yes / no | 1.163 | 0.833-1.623 | .375 | 1.111 | 0.810-1.524 | .515 | | 1.112 | | 0.756-1.637 | | .590 | |
| Tumor size (cm) | ≥5 / <5 | 4.180 | 2.798-6.244 | **<.001** | 2.259 | 1.633-3.123 | **<.001** | | 2.631 | | 1.725-4.010 | | **<.001** | |
| Histological grade | III-IV / I-II | 1.295 | 0.887-1.891 | .180 | 1.281 | 0.902-1.821 | .167 | | 1.858 | | 1.147-3.011 | | **.012** | |
| Pathological satellite | Yes / no | 1.848 | 1.334-2.561 | **<.001** | 2.327 | 1.710-3.165 | **<.001** | | 1.893 | | 1.306-2.744 | | **.001** | |
| Microvascular Invasion | Yes / no | 2.299 | 1.668-3.168 | **<.001** | 2.868 | 2.112-3.895 | **<.001** | | 2.408 | | 1.669-3.475 | | **<.001** | |
| HBsAg | Positive / negative | 1.171 | 0.816-1.681 | .391 | 1.072 | 0.766-1.499 | .686 | | 1.122 | | 0.757-1.664 | | .566 | |
| TNM stage | II+III / I | 5.587 | 3.323-9.392 | **<.001** | 3.671 | 2.452-5.494 | **<.001** | | 4.251 | | 2.429-7.440 | | **<.001** | |
| Protein level of UCK2 | High / low | 2.237 | 1.608-3.112 | **<.001** | 2.188 | 1.606-2.982 | **<.001** | | 2.115 | | 1.446-3.094 | | **<.001** | |
| **Multivariate analysis** | | | | | | | |  | |  | |  | |  |
| AFP (μg/L) | ≥20 / <20 | 1.191 | 0.820-1.728 | .359 | 1.190 | 0.845-1.675 | .319 | | 1.105 | | 0.718-1.700 | | .651 | |
| Tumor size (cm) | ≥5 / <5 | 2.093 | 1.146-3.820 | **.016** | 1.043 | 0.644-1.689 | .865 | | 1.093 | | 0.595-2.009 | | .774 | |
| Histological grade | III-IV / I-II | - | - | - | - | - | - | | 1.332 | | 0.806-2.201 | | .263 | |
| Pathological satellite | Yes / no | 1.560 | 0.874-2.786 | .133 | 1.600 | 0.907-2.825 | .104 | | 1.656 | | 0.870-3.155 | | .124 | |
| Microvascular Invasion | Yes / no | 2.011 | 1.130-3.580 | **.018** | 2.309 | 1.307-4.077 | **.004** | | 2.180 | | 1.144-4.154 | | **.018** | |
| TNM stage | II+III / I | 2.331 | 1.036-5.242 | **.041** | 2.877 | 1.497-5.528 | **.002** | | 2.868 | | 1.216-6.768 | | **.016** | |
| Protein level of UCK2 | High / low | 1.678 | 1.173-2.402 | **.005** | 1.742 | 1.249-2.431 | **.001** | | 1.609 | | 1.066-2.427 | | **.024** | |

AFP, alpha-fetoprotein; TNM, tumor-node-metastasis; HR, hazard ratio; 95% CI, 95% confidence interval. A value of *P* < 0.05 was considered to be significant.
