# Supplementary material for: Non-metabolic role of UCK2 links EGFR-AKT pathway activation to metastasis enhancement in hepatocellular carcinoma
Source: Oncogenesis. 2020 Dec 4;9(12):103. doi: 10.1038/s41389-020-00287-7 (PMC7718876; doi:10.1038/s41389-020-00287-7)
Supplement: Supplementary file 6 — Table S5 [file 41389_2020_287_MOESM6_ESM.doc]

**Supplementary table 5. Significantly altered phosphorylated proteins caused by UCK2^WT^ or UCK2^D62A^** in the cancer signaling phospho-antibody array. Related to Figure 4

| **Protein name** | **Phosphorylation Site** | **LV-NC** | | | **LV-UCK2^WT^** | | | **LV-UCK2^D62A^** | | | **LV-UCK2^WT^ vs LV-NC** | **LV-UCK2^D62A^ vs LV-NC** |
| --- | --- | --- | --- | --- | --- | --- | --- | --- | --- | --- | --- | --- |
|  |  | **phospho** | **unphospho** | **phospho_ratio_** | **phospho** | **unphospho** | **phospho_ratio_** | **phospho** | **unphospho** | **phospho_ratio_** |  |  |
| AKT1 | Ser473 | 86.00 | 387.50 | 0.22 | 163.83 | 292.40 | 0.56 | 158.67 | 296.00 | 0.54 | **2.55** | **2.45** |
| BAD | Ser112 | 80.17 | 334.67 | 0.24 | 120.50 | 344.00 | 0.35 | 131.83 | 328.83 | 0.40 | 1.46 | **1.67** |
| BRCA1 | Ser1423 | 97.00 | 358.00 | 0.27 | 65.60 | 381.40 | 0.17 | 78.33 | 416.20 | 0.19 | **0.63** | 0.70 |
| ELK1 | Ser383 | 80.67 | 366.67 | 0.22 | 141.40 | 317.67 | 0.45 | 126.67 | 287.40 | 0.44 | **2.05** | **2.00** |
| JAK2 | Tyr221 | 141.80 | 429.20 | 0.33 | 100.60 | 478.00 | 0.21 | 84.60 | 466.20 | 0.18 | **0.64** | **0.55** |
| JUN | Ser73 | 141.33 | 544.33 | 0.26 | 86.33 | 534.60 | 0.16 | 76.40 | 587.20 | 0.13 | **0.62** | **0.50** |
| JUN | Ser243 | 67.00 | 561.00 | 0.12 | 98.50 | 529.00 | 0.19 | 99.00 | 602.40 | 0.16 | **1.58** | 1.33 |
| JUNB | Ser259 | 77.80 | 277.80 | 0.28 | 99.83 | 226.33 | 0.44 | 108.00 | 231.40 | 0.47 | **1.57** | **1.68** |
| KRT18 | Ser33 | 61.00 | 132.20 | 0.46 | 89.30 | 92.80 | 0.96 | 76.67 | 112.60 | 0.68 | **2.09** | 1.48 |
| MAPK6 | Ser189 | 583.40 | 426.25 | 1.37 | 668.40 | 364.50 | 1.83 | 682.83 | 332.33 | 2.05 | 1.34 | **1.50** |
| MYC | Ser373 | 115.83 | 312.20 | 0.37 | 157.67 | 225.80 | 0.70 | 176.80 | 234.60 | 0.75 | **1.89** | **2.03** |
| NFKBIA | Ser32/Ser36 | 486.17 | 423.00 | 1.15 | 590.40 | 303.60 | 1.94 | 621.00 | 298.60 | 2.08 | **1.69** | **1.81** |
| PDPK1 | Ser241 | 120.33 | 326.60 | 0.37 | 165.67 | 252.40 | 0.66 | 153.20 | 302.50 | 0.51 | **1.78** | 1.38 |
| RELA | Thr254 | 63.67 | 275.50 | 0.23 | 90.67 | 240.17 | 0.38 | 82.80 | 291.50 | 0.28 | **1.65** | 1.22 |
| STAT6 | Tyr641 | 64.33 | 269.33 | 0.24 | 87.17 | 234.67 | 0.37 | 98.17 | 242.83 | 0.40 | **1.54** | **1.67** |
| YWHAZ | Ser58 | 126.00 | 425.67 | 0.30 | 93.33 | 494.17 | 0.19 | 99.00 | 480.50 | 0.21 | **0.63** | 0.70 |
