# Supplementary material for: Non-metabolic role of UCK2 links EGFR-AKT pathway activation to metastasis enhancement in hepatocellular carcinoma
Source: Oncogenesis. 2020 Dec 4;9(12):103. doi: 10.1038/s41389-020-00287-7 (PMC7718876; doi:10.1038/s41389-020-00287-7)
Supplement: Supplementary file 7 — Table S6 [file 41389_2020_287_MOESM7_ESM.doc]

**Supplementary table 6. The changes in phosphorylation intensity of receptor tyrosine kinases caused by UCK2^WT^ or UCK2^D62A^** in the cancer signaling phospho-antibody array. Related to Figure 5

| **Protein name** | **Phosphorylation Site** | **LV-NC** | | | **LV-UCK2^WT^** | | | **LV-UCK2^D62A^** | | | **LV-UCK2^WT^ vs LV-NC** | **LV-UCK2^D62A^ vs LV-NC** |
| --- | --- | --- | --- | --- | --- | --- | --- | --- | --- | --- | --- | --- |
|  |  | **phospho** | **unphospho** | **phospho_ratio_** | **phospho** | **unphospho** | **phospho_ratio_** | **phospho** | **unphospho** | **phospho_ratio_** |  |  |
| EGFR | Tyr1110 | 74.83 | 274.33 | 0.27 | 127.20 | 479.00 | 0.27 | 128.50 | 489.60 | 0.26 | 1.00 | 0.96 |
| ERBB2 | Tyr877 | 81.00 | 267.67 | 0.30 | 76.33 | 242.20 | 0.32 | 79.50 | 260.17 | 0.31 | 1.07 | 1.03 |
| FGFR1 | Tyr154 | 114.00 | 376.83 | 0.30 | 112.60 | 382.50 | 0.29 | 112.60 | 381.40 | 0.30 | 0.97 | 1.00 |
| IGF1R | Tyr1161 | 78.50 | 230.33 | 0.34 | 76.60 | 218.33 | 0.35 | 74.00 | 206.17 | 0.36 | 1.03 | 1.06 |
| KDR | Tyr951 | 74.00 | 373.33 | 0.20 | 75.00 | 371.00 | 0.20 | 66.83 | 355.50 | 0.19 | 1.00 | 0.95 |
| KIT | Tyr721 | 110.67 | 472.00 | 0.23 | 100.00 | 440.00 | 0.23 | 99.33 | 420.40 | 0.24 | 1.00 | 1.04 |
| MET | Tyr1349 | 118.60 | 361.67 | 0.33 | 115.67 | 376.00 | 0.31 | 100.83 | 302.80 | 0.33 | 0.94 | 1.00 |
| NTRK2 | Tyr515 | 103.33 | 550.80 | 0.19 | 103.17 | 506.00 | 0.20 | 110.50 | 518.33 | 0.21 | 1.05 | 1.11 |
| PDGFRB | Tyr751 | 100.67 | 311.60 | 0.32 | 109.00 | 293.00 | 0.37 | 107.00 | 299.50 | 0.36 | 1.16 | 1.13 |
