# Supplementary material for: Non-metabolic role of UCK2 links EGFR-AKT pathway activation to metastasis enhancement in hepatocellular carcinoma
Source: Oncogenesis. 2020 Dec 4;9(12):103. doi: 10.1038/s41389-020-00287-7 (PMC7718876; doi:10.1038/s41389-020-00287-7)
Supplement: Supplementary file 8 — Table S7 [file 41389_2020_287_MOESM8_ESM.doc]

Supplementary table 7. Primer pairs for qRT-PCR.

| **Gene** | **Forward (5’-3’)** | **Reverse (5’-3’)** |
| --- | --- | --- |
| UCK2 (human) | CTGAGCCAGGATAGCTTCTACC | CATACACGGGGATCTGGACTG |
| EGFR (human) | AGGCACGAGTAACAAGCTCAC | ATGAGGACATAACCAGCCACC |
| 18S (human) | GGAGAGGGAGCCTGAGAAACG | TTACAGGGCCTCGAAAGAGTCC |
| β-actin (human) | GGGAAATCGTGCGTGACATTAAG | TGTGTTGGCGTACAGGTCTTTG |
