# Supplementary figures and images for: Non-metabolic role of UCK2 links EGFR-AKT pathway activation to metastasis enhancement in hepatocellular carcinoma
Source: Oncogenesis. 2020 Dec 4;9(12):103. doi: 10.1038/s41389-020-00287-7 (PMC7718876; doi:10.1038/s41389-020-00287-7)

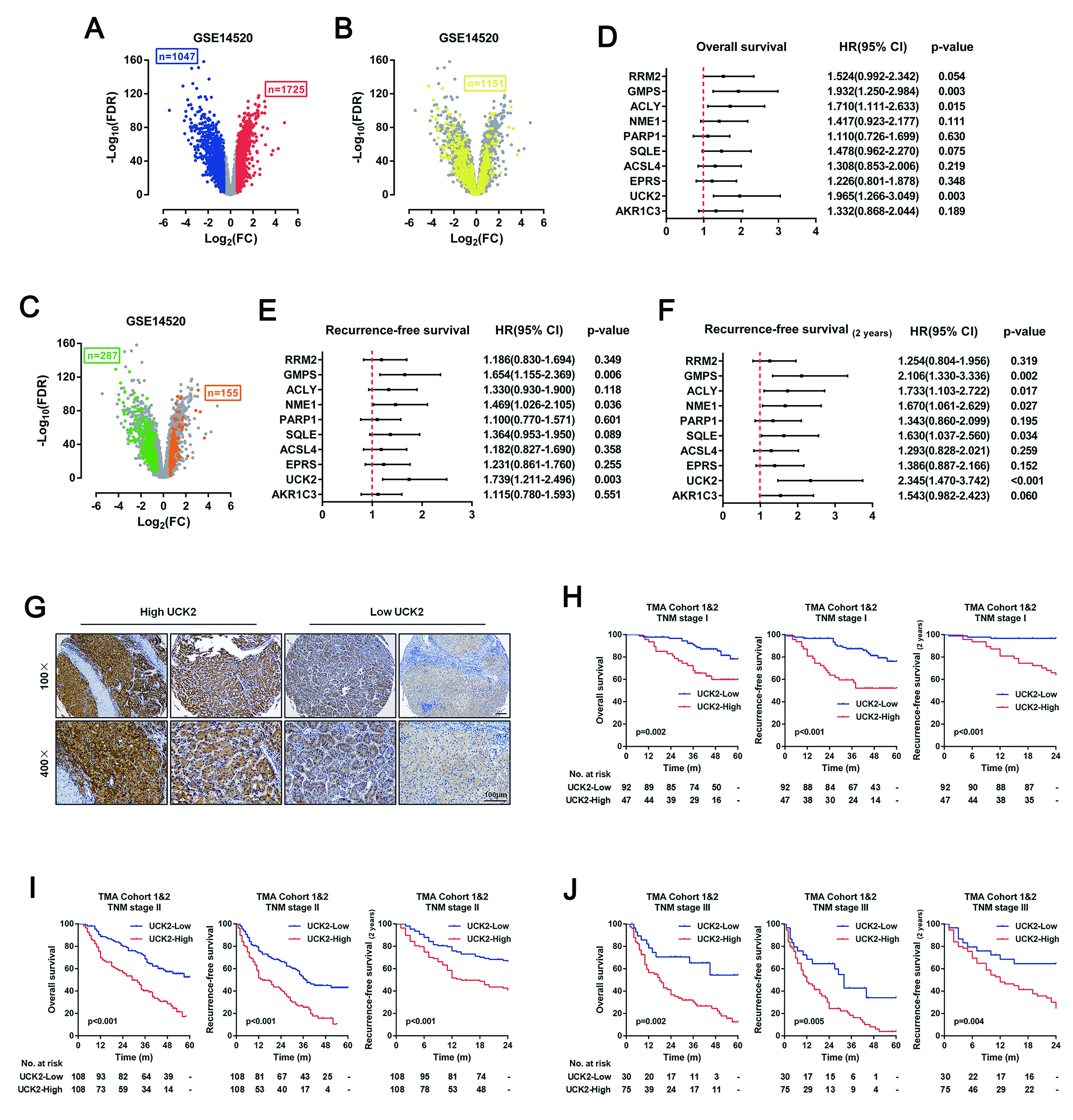

Supplement: Supplementary file 9 — Figure S1 [file 41389_2020_287_MOESM9_ESM.tif]

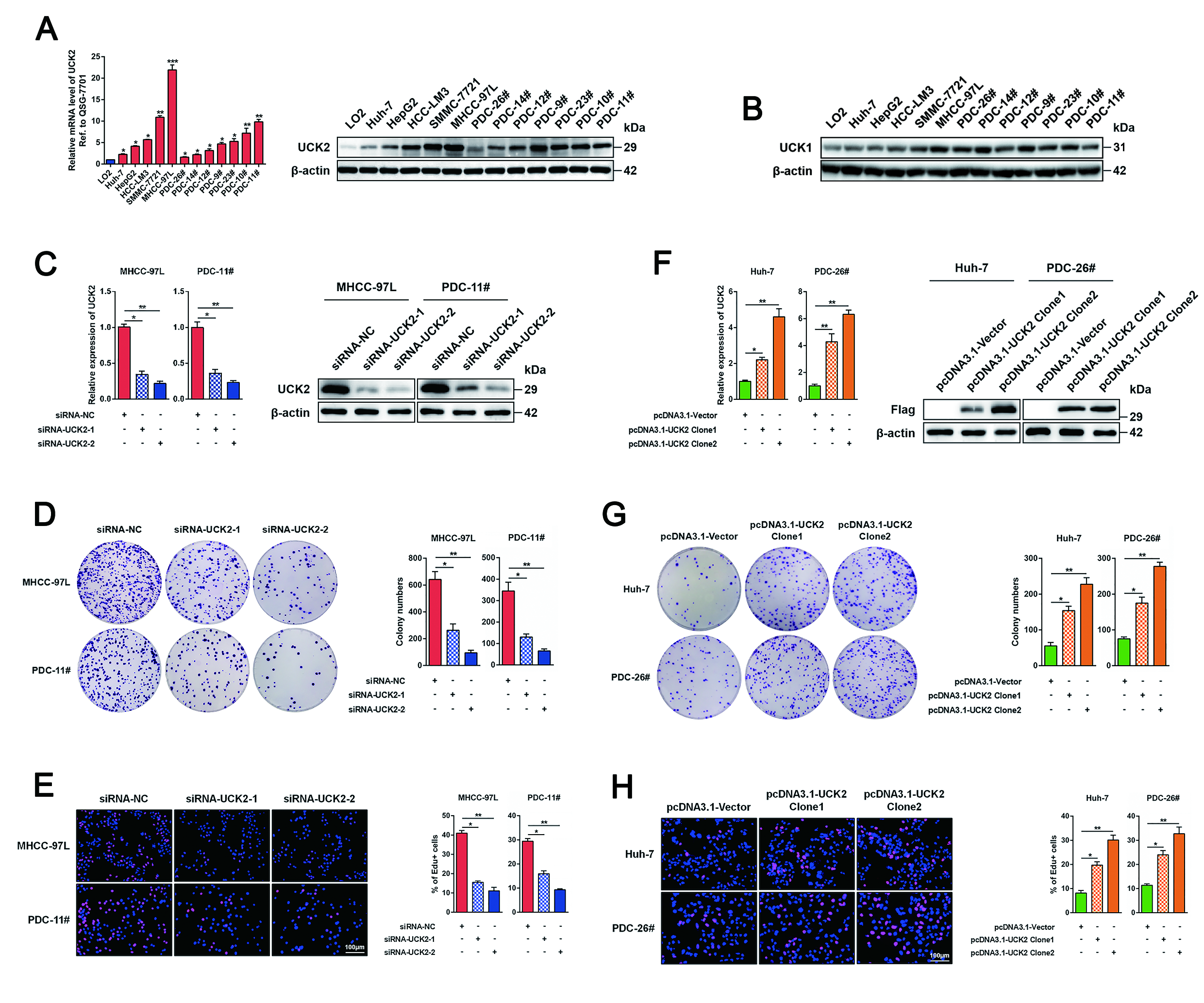

Supplement: Supplementary file 10 — Figure S2 [file 41389_2020_287_MOESM10_ESM.tif]

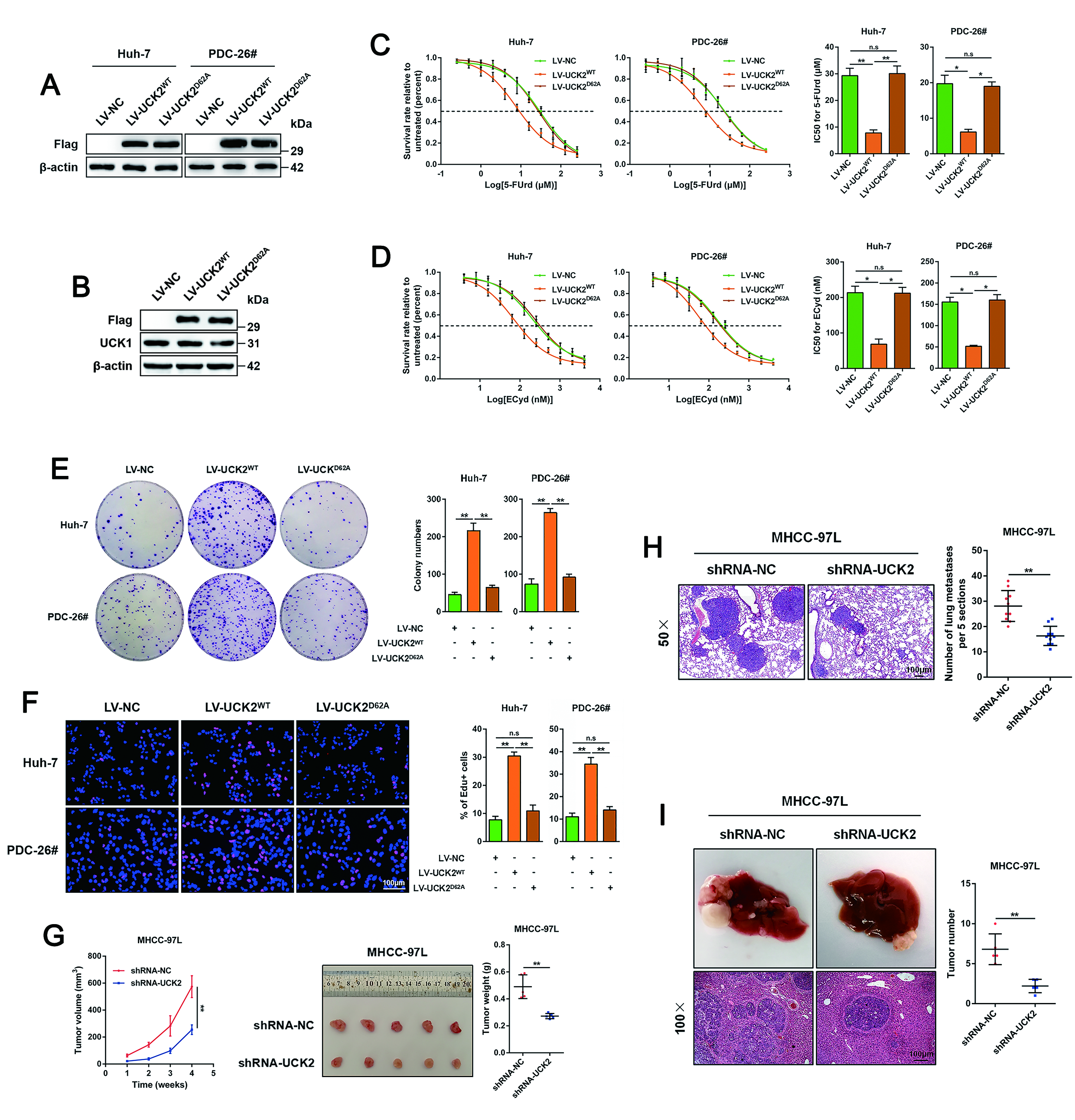

Supplement: Supplementary file 11 — Figure S3 [file 41389_2020_287_MOESM11_ESM.tif]

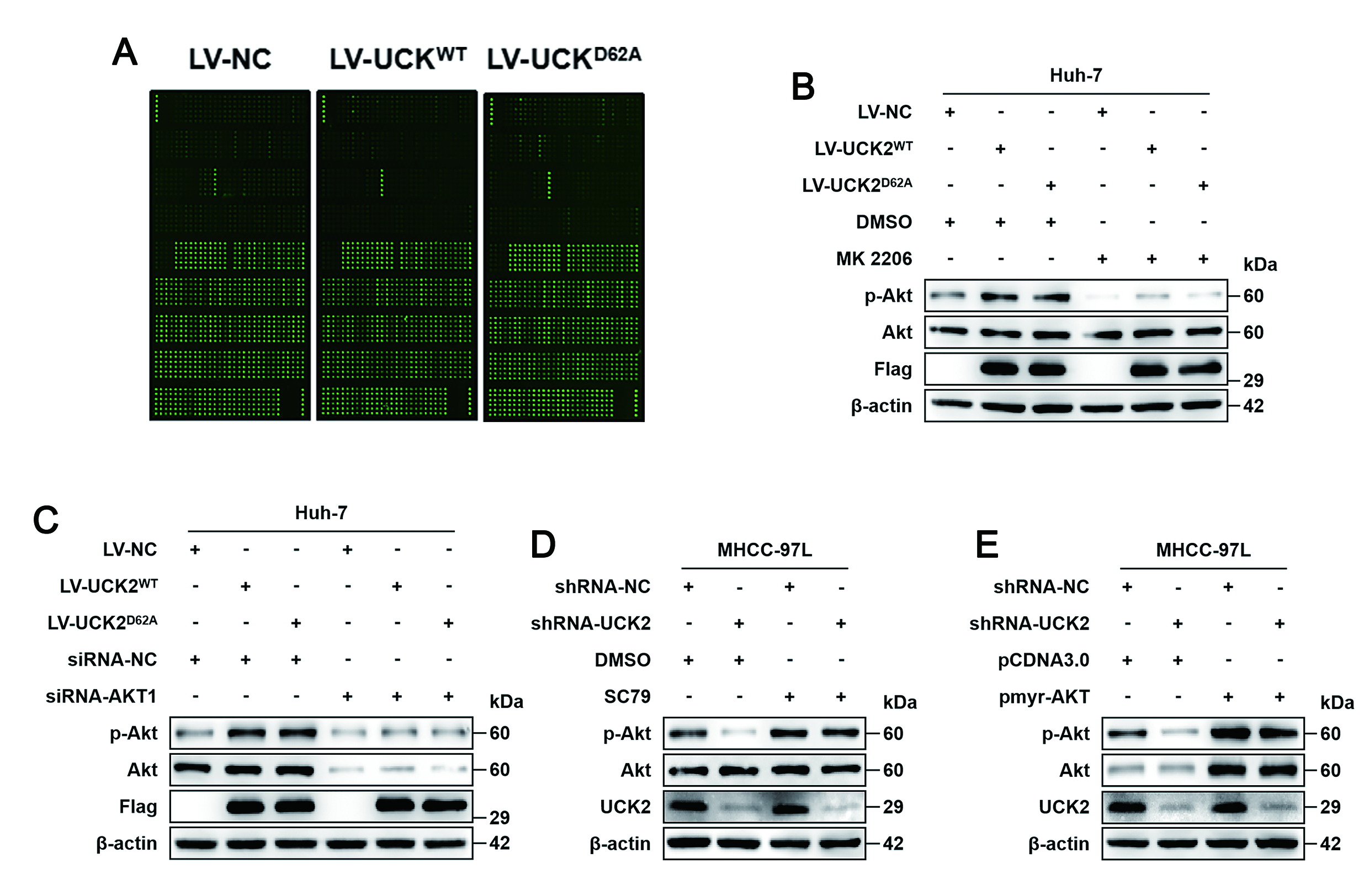

Supplement: Supplementary file 12 — Figure S4 [file 41389_2020_287_MOESM12_ESM.tif]

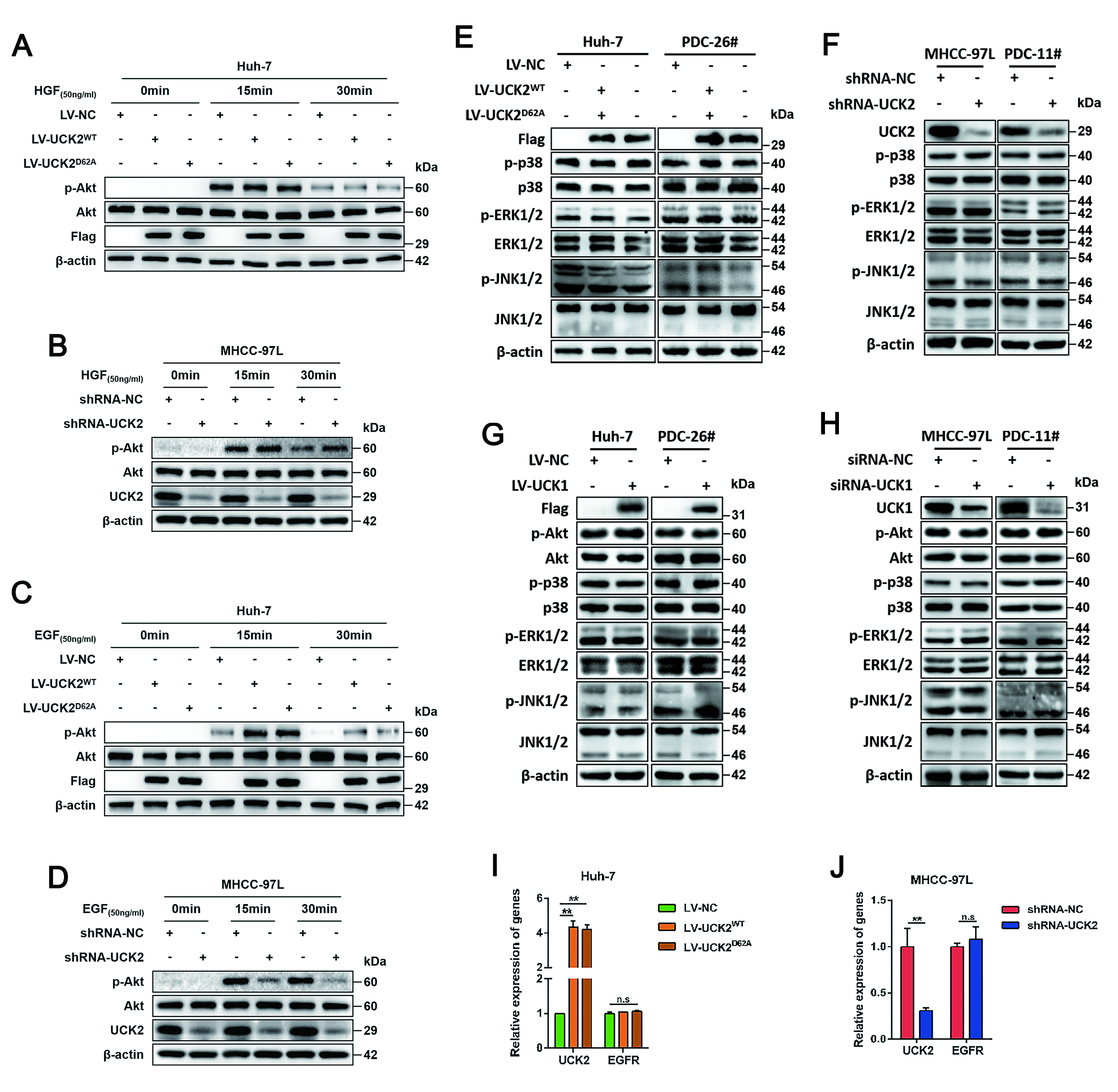

Supplement: Supplementary file 13 — Figure S5 [file 41389_2020_287_MOESM13_ESM.tif]

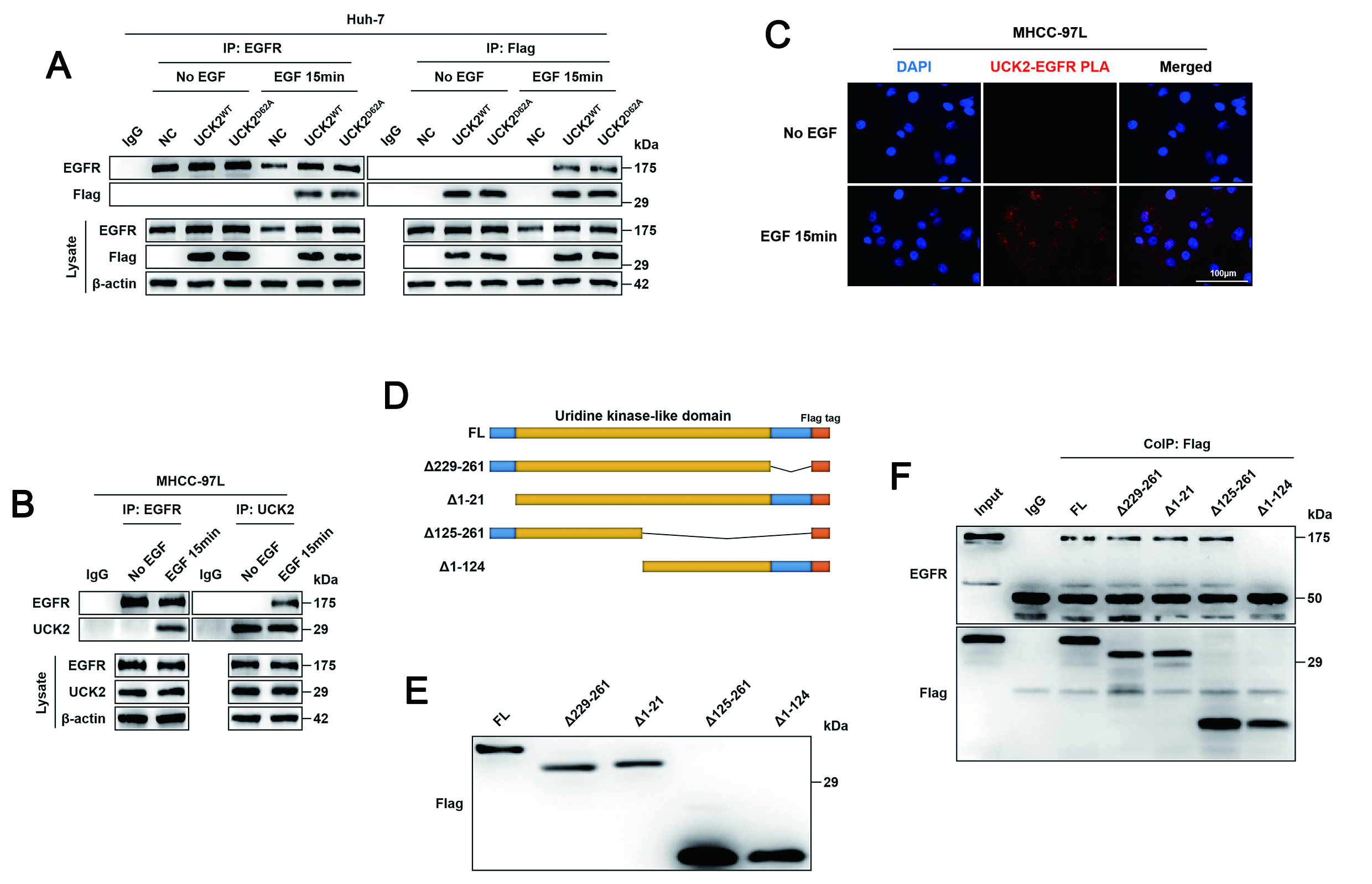

Supplement: Supplementary file 14 — Figure S6 [file 41389_2020_287_MOESM14_ESM.tif]

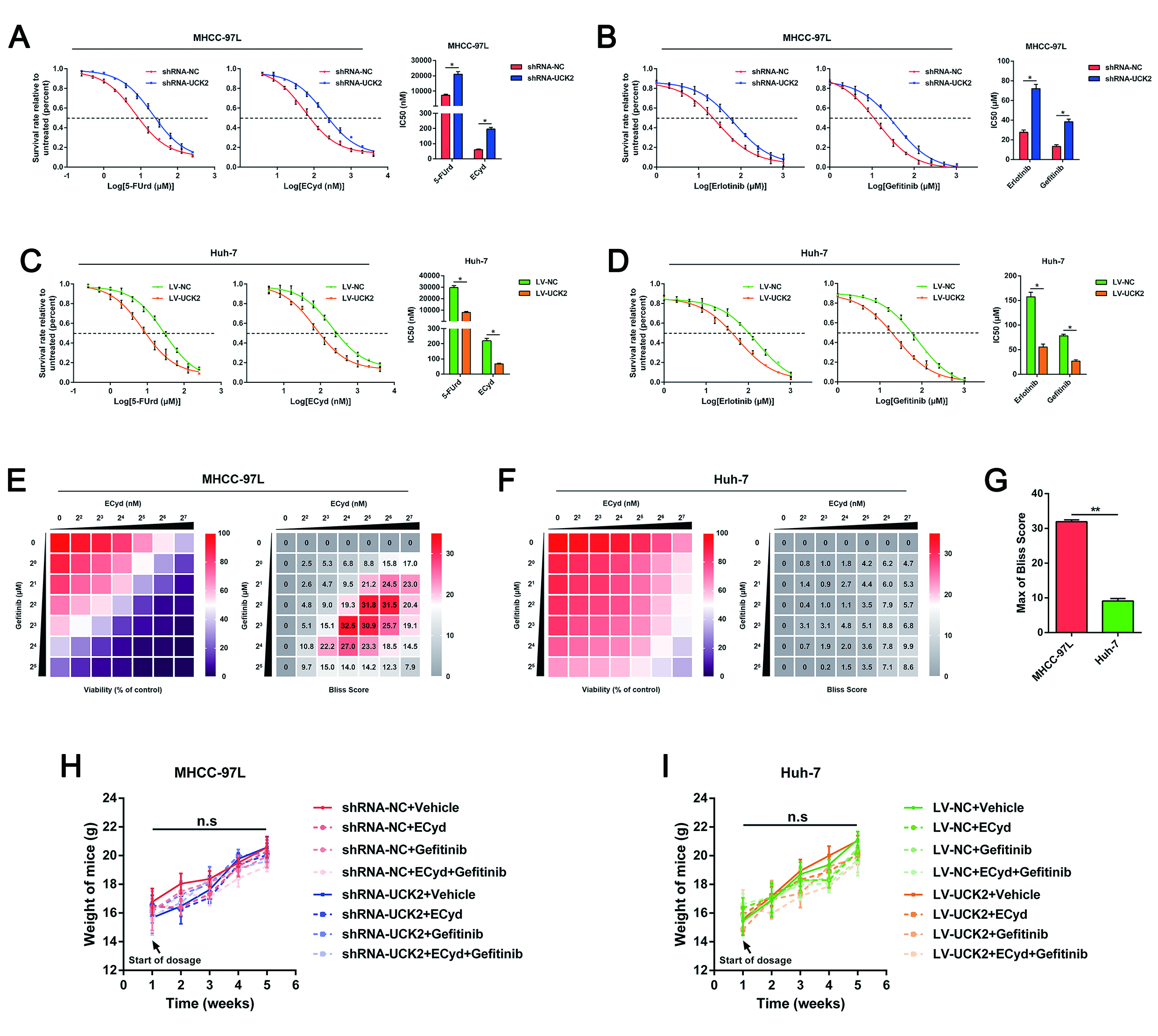

Supplement: Supplementary file 15 — Figure S7 [file 41389_2020_287_MOESM15_ESM.tif]

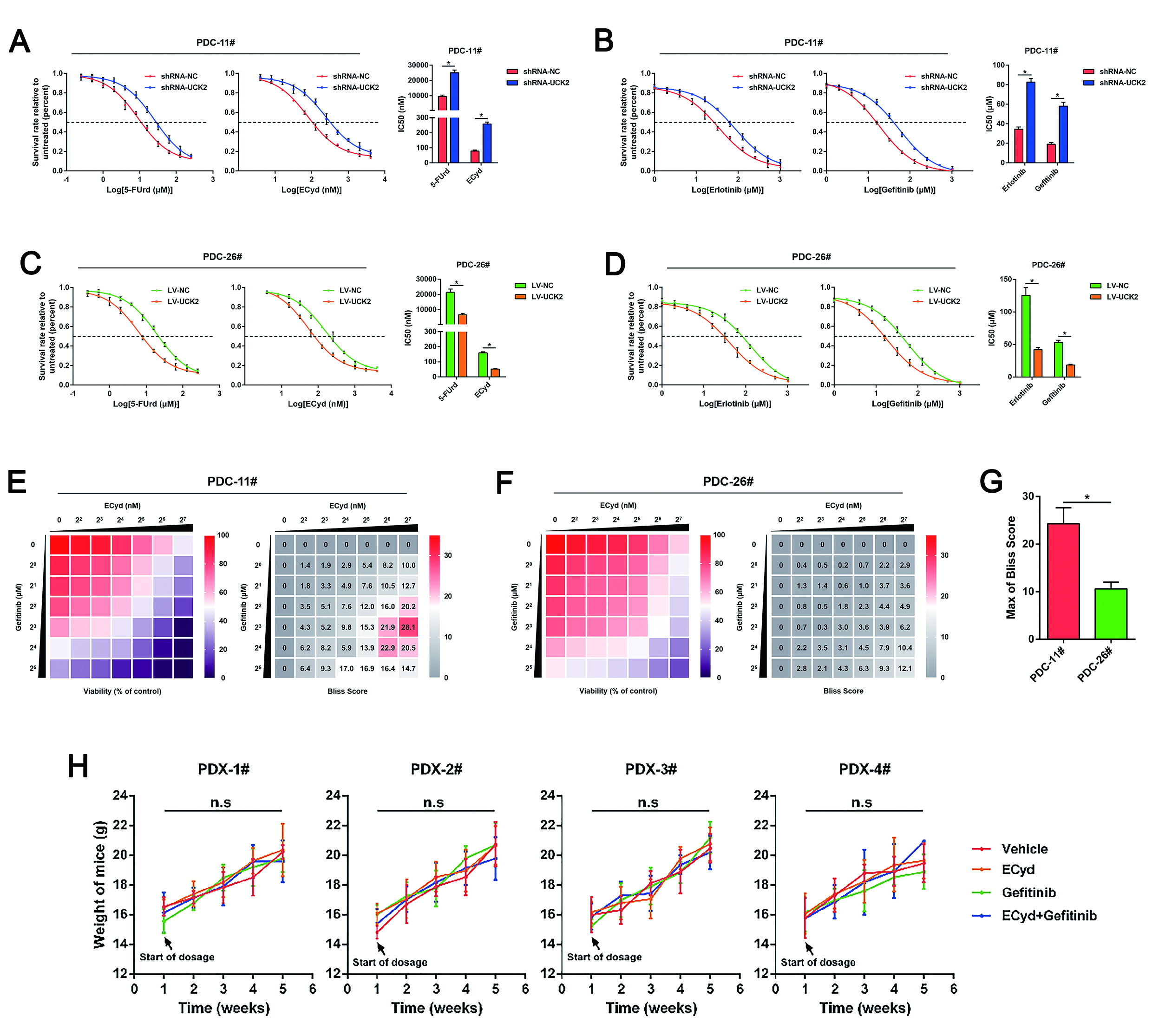

Supplement: Supplementary file 16 — Figure S8 [file 41389_2020_287_MOESM16_ESM.tif]
